# Nanosecond pulse effectively ablated hepatocellular carcinoma with alterations in the gut microbiome and serum metabolites

**DOI:** 10.3389/fphar.2023.1163628

**Published:** 2023-05-10

**Authors:** Yawen Zou, Ying Sun, Xinhua Chen, Liangjie Hong, Gang Dong, Xiwen Bai, Haiyu Wang, Benchen Rao, Zhigang Ren, Zujiang Yu

**Affiliations:** ^1^ Department of Infectious Diseases, The First Affiliated Hospital of Zhengzhou University, Zhengzhou, China; ^2^ Key Laboratory of Pulsed Power Translational Medicine of Zhejiang Province, Hangzhou, China; ^3^ Gene Hospital of Henan Province, Precision Medicine Center, The First Affiliated Hospital of Zhengzhou University, Zhengzhou, China; ^4^ Department of Ultrasound, The First Affiliated Hospital of Zhengzhou University, Zhengzhou, China; ^5^ Nanchang University Queen Marry School, Nanchang, Jiangxi, China

**Keywords:** hepatocellular carcinoma, nanosecond pulsed electric fields, gut microbiome, serum metabolites, ablation

## Abstract

**Background:** Hepatocellular carcinoma (HCC) is the third leading cause of cancer-related death in the world. Nanosecond pulsed electric fields (nsPEFs) have emerged as a new treatment for cancer. This study aims to identify the effectiveness of nsPEFs in the treatment of HCC and analyze the alterations in the gut microbiome and serum metabonomics after ablation.

**Methods:** C57BL/6 mice were randomly divided into three groups: healthy control mice (*n* = 10), HCC mice (*n* = 10), and nsPEF-treated HCC mice (*n* = 23). Hep1-6 cell lines were used to establish the HCC model *in situ*. Histopathological staining was performed on tumor tissues. The gut microbiome was analyzed by 16S rRNA sequencing. Serum metabolites were analyzed by liquid chromatography–mass spectrometry (LC-MS) metabolomic analysis. Spearman’s correlation analysis was carried out to analyze the correlation between the gut microbiome and serum metabonomics.

**Results:** The fluorescence image showed that nsPEFs were significantly effective. Histopathological staining identified nuclear pyknosis and cell necrosis in the nsPEF group. The expression of CD34, PCNA, and VEGF decreased significantly in the nsPEF group. Compared with normal mice, the gut microbiome diversity of HCC mice was increased. Eight genera including *Alistipes* and *Muribaculaceae* were enriched in the HCC group. Inversely, these genera decreased in the nsPEF group. LC-MS analysis confirmed that there were significant differences in serum metabolism among the three groups. Correlation analysis showed crucial relationships between the gut microbiome and serum metabolites that are involved in nsPEF ablation of HCC.

**Conclusion:** As a new minimally invasive treatment for tumor ablation, nsPEFs have an excellent ablation effect. The alterations in the gut microbiome and serum metabolites may participate in the prognosis of HCC ablation.

## 1 Introduction

Hepatocellular carcinoma (HCC) is the fourth leading cause of cancer-related death worldwide. In Western countries such as the United Kingdom and the United States, the incidence of HCC related to non-alcoholic fatty liver disease (NAFLD) has been increasing in recent years (Huang et al., 2021). Due to the high prevalence of hepatitis B virus (HBV) infection and HBV-induced cirrhosis in China, the incidence of HCC is high. In 2020, the number of newly diagnosed cases of HCC in China was 0.41 million, and the mortality rate of HCC ranked second among all types of cancer (Cao et al., 2021). Finding effective therapeutic strategies for HCC has become an important scientific research problem to be solved urgently.

Pulsed electric fields (PEFs) can break through the cell membrane. Since it was discovered in the 1970s, it has been used in a variety of biological studies, such as gene transfection *in vitro* (Breton and Mir, 2012), gene therapy (Henshaw et al., 2008), electrochemical therapy, and cell fusion (Li et al., 2018). With the deepening of research, the PEF gradually showed its advantages in tumor treatment. Aleksander Kiełbik et al. found that the cytoskeleton and fluidity of prostate cancer cells changed significantly when they were exposed to high-frequency nsPEFs (Kielbik et al., 2021), which led to enhancement of cell membrane permeability, formation of tiny nanopores on the membrane (Pakhomov et al., 2007), change of membrane potential, release of cytochrome C, and mobilization of calcium ions (Ford et al., 2010), resulting in apoptosis or necrosis of the cells. The use of electrical pulses for tumor cell death technology is non-thermal, so it does not damage blood vessels. In addition, the effect of nsPEFs on activating antitumor immune response has also been reported by several research teams (Xu et al., 2018; Yimingjiang et al., 2020).

Various research groups (Chen et al., 2017; Guo et al., 2018; Xu et al., 2018; Nuccitelli, 2019) have previously confirmed through *in vivo* and *in vitro* experiments that nanosecond pulse has good tissue selectivity, and its effectiveness and safety have been confirmed in the treatment of tumors close to the blood vessels. The most striking effect of nanosecond pulse is the immune activation effect, known as nano-pulse stimulation (NPS). Nanosecond pulse can induce apoptosis through a non-thermogenic electric field energy transmembrane into the nucleus while retaining tumor antigens on the cell membrane, attracting immune recognition, making macrophages infiltrate and differentiate into tumor-inhibiting phenotypes, increasing the number of CD8 + T cells and enhancing the killing ability of CD8 + T cells to inhibit HCC recurrence and metastasis, and acting as an immune modulator. Combined drugs enhance the effect of comprehensive treatment.

Microecological changes are involved in the development of many diseases, such as latent autoimmune diabetes in adults (LADA) (Fang et al., 2021), colorectal cancer (CRC) (Feng et al., 2015), and chronic kidney disease (CKD) (Ren et al., 2020). Our previous studies have also shown that the predictive model of the gut microbiome also has a strong diagnostic ability for HCC (Ren et al., 2019). This shows that changes in the gut microbiome are of great significance in the progression of HCC. Herein, the scientific question that we are very concerned about is whether the ablation of HCC by nsPEFs can produce changes in the gut microbiome.

Host metabolism will be affected by pathophysiological changes in the process of disease progression, and abnormal metabolism will accelerate the occurrence of abnormal pathological processes. Therefore, metabonomics is also widely used in the study of many diseases (Weiss and Kim, 2011; Ussher et al., 2016; McGlinchey et al., 2022). The liver is the largest digestive organ in the body and is also involved in mediating many metabolic reactions, such as protein, fat, and carbohydrate. Existing studies have shown that metabolic reprogramming plays an indispensable role in the occurrence and development of HCC. Compared with the normal tissue around the focus, the urea cycle in HCC tissue is significantly inhibited, aerobic glycolysis is more obvious (Wang et al., 2022), and lipid metabolism is also more active (Hall et al., 2021). Based on the results of previous metabonomic studies, we explore the changes in metabolites in HCC tissue after nsPEF treatment.

In this study, we analyzed the gut microbiome and serum metabolites of 10 normal, 10 *in situ* HCC, and 23 after nsPEF ablation mice. The characteristics of the gut microbiome and serum metabolites of HCC mice after nsPEF treatment were clarified. Then, we discussed the effect of nanosecond pulse on the body after nsPEF treatment. This is of great significance for the follow-up application of nsPEFs in clinical research and disease treatment.

## 2 Materials and methods

### 2.1 Cell lines and cell culture

Human HCC cell lines Hep1-6 were amiably provided by Zhejiang Academy of Medical Sciences, incubated at 37°C in a 5% CO_2_ incubator, and cultured in DMEM supplemented with 10% FBS (Gibco, Carlsbad, CA, United States). The cell lines were mycoplasma-negative.

### 2.2 Animals

C57BL/6 mice were purchased from Hangzhou Medical College at 7–8 weeks of age. All the mice were healthy, had no other underlying diseases, and the average weight was about 25 g. The mice were cared for carefully by experienced experimental breeders. Under the same environmental conditions in the animal housing room, the mice were provided with the same formula diet, clear water, and adequate light for 12 h per day. After anesthesia, the abdominal cavity was opened up to 1 cm, and 1 × 10^6^ Hep1-6 tumor cells were injected under the left liver capsule to establish the tumor *in situ*. The animal experiment was approved by the experimental Animal Welfare Ethics Committee of the Zhejiang Animal Experiment Center (ZJCLA-IACUC-20040072).

### 2.3 Animal model establishment

Forty-three C57BL/6 tumor-bearing mice were randomly divided into three groups: healthy control mice (C group, n= 10); HCC mice (M group, n = 10); and nsPEF-treated HCC mice (N group, n = 23). The C group was given adequate water and feed *ad libitum*.

The mice in the M group (five mice in each cage) were weighed before anesthesia and intraperitoneal injection. The anesthetized mice were marked with ear labels, and the original data were recorded. In the supine position, the mice were fixed upward on the adhesive board with a non-woven medical tape (there should be no confusion between mice in each cage). The blown cell suspension was extracted from 10 ul/30 WIU using a 500-ul syringe and injected into the left lobe of the liver. The mice were imaged *in vivo* after 2 weeks to ensure that the HCC model was successfully established. All mice were fed the same feed, provided the same water, and maintained in the same living environment and temperature. The mice were starved for one night before collecting feces and blood samples to eliminate interference factors. Blood samples were obtained by collecting blood from the inferior vena cava after anesthetic laparotomy. Fecal samples were collected from dry feces from the anus, about 1–1.5 cm. The serum, feces, and histological samples were collected, and the mice were euthanized. The HCC model was established by the same method as observed in the N group, which was different from that in the M group, in that the N group needed nanosecond pulse ablation for the local lesion. Similarly, the same samples were preserved before execution 3 days after ablation.

Liver histological samples were immediately soaked in formalin solution *in vitro* and fixed for more than 48 h; they were then were dehydrated, embedded, and sectioned. The normal control group was stained only with hematoxylin and eosin (HE), while the model group and treatment group were stained with CD34, VEGF, PCNA, and TUNEL.

### 2.4 Procedures of nsPEF ablation

The ablation instrument is obtained from the Key Laboratory of Pulsed Power Translational Medicine of Zhejiang Province. The pulse parameters were set as follows: pulse number: 50 pulses per electrode; electric voltage: 15 kV; and duration: 300 ns. A pair of needle electrodes was implanted into the tumor center to deliver an electric field. They were electrically insulated 0.5 cm from the tip. After the instrument is electrified, an electric field is generated in the center of the tumor, and an oval non-thermal ablation zone is formed around the tip of the needle.

### 2.5 Sample collection and processing

We have developed a strict sample collection protocol to reduce the interference caused by improper operation. About 4 ml blood was collected from the inferior vena cava of each mouse and directly collected in the EDTA blood vessel containing the anticoagulant. The supernatant was collected in a 1.5-ml EP tube after centrifugation 5–6 times. The supernatant was stored in the refrigerator at -80°C until metabolomics analysis was performed.

Fecal samples about 1–2 cm long were collected from the anus of the mice and stored in EP tubes. After marking, they were immediately transferred to a cryogenic refrigerator at -80°C until the 16S rRNA MiSeq sequencing analysis was conducted.

### 2.6 Serum metabolic detection and analysis

The serum samples were detected by ultrahigh-performance liquid chromatography–mass spectrometry (UPLC-MS) in positive and negative modes, respectively, to obtain the mass spectrometry (MS) and tandem mass spectrometry (MS/MS) information of metabolites. The quality control sample was mixed to evaluate the repeatability and stability of the UPLC-MS analysis process. The data were preprocessed by Progenesis QI software (Waters Corporation, Milford, United States). The metabolites were annotated in combination with the self-built databases, HMDB and Metlin, to obtain the metabolite list and data matrix. Finally, differential metabolites were screened by the *t*-test and variable importance in projection (VIP), and the biological information of differential metabolites was further visualized. Finally, the differential metabolites were obtained by statistical analysis.

### 2.7 Microflora analysis of 16S rRNA sequencing

The fecal samples were collected in EP tubes. According to our previous research methods, DNA extraction was completed by using a genomic DNA extraction kit (Ren et al., 2019). After DNA extraction, PCR amplification, and DNA library construction, the amplified sequences were sequenced using the Illumina MiSeq platform (Shanghai MoBIO Biomedical Technology Co. Ltd.). Furthermore, the original data were preprocessed, and the UPARSE pipeline and RDP classifier version 2.6 were used to perform operational taxonomic unit (OTU) clustering and species annotation, respectively. Species diversity analysis, species structure analysis, and species difference analysis were carried out based on OTUs, and the optimal OTU combination was identified in the random forest model through five-fold cross-validation.

### 2.8 Gut microbiome–serum metabolite correlation analysis

Spearman’s correlation analysis was carried out to calculate the correlation between the gut microbiome and serum metabolites in groups C and M, M and N, and C and N.

### 2.9 Statistical analysis

SPSS v. 20.0 (IBM Corp., Armonk, NY, United States) and GraphPad Prism 6 were used to analyze the data. The statistical significance of the differences among the three groups was calculated. The Wilcoxon rank-sum test and Student’s *t*-test were conducted to compare the continuous variables between two groups. Categorical variables were compared using Fisher’s exact test. Correlation analysis was conducted using Spearman’s rank test. The Kruskal–Wallis test was used to compare the overall differences among the three groups. The Mann–Whitney U test was used for the comparison of two groups. *p* < 0.05 (two-sided) indicated statistical significance.

## 3 Results

### 3.1 Study design

**FIGURE 1:**
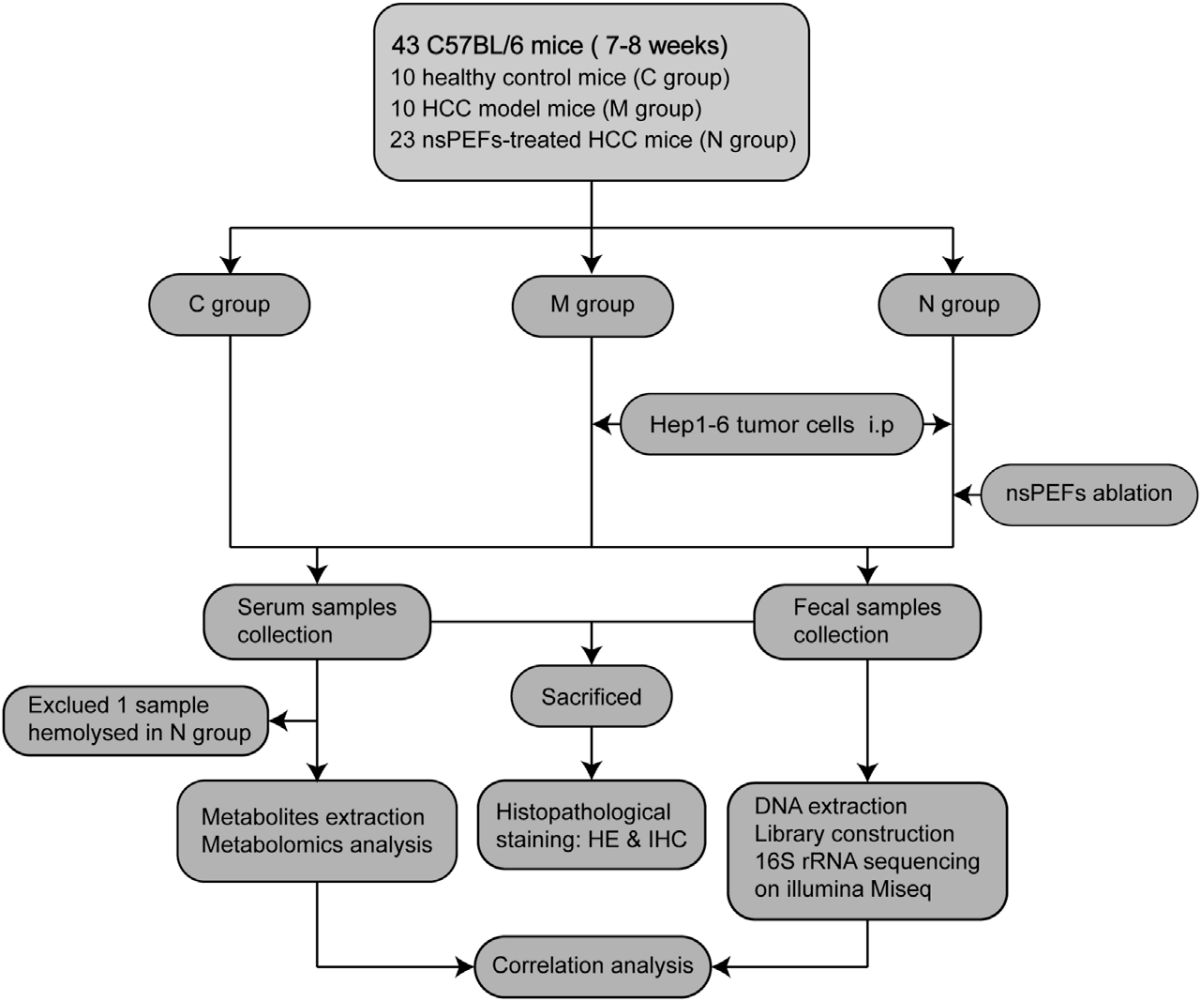
Study design and flow diagram. A total of 43 mice were randomly divided into three groups: healthy control mice (C group, n = 10), HCC mice (M group, n = 10), and nsPEF-treated HCC mice (N group, n = 23). Serum samples were collected from 42 mice (excluding one sample hemolysis), and fecal samples were collected from all 43 mice. HCC: hepatocellular carcinoma; HE: hematoxylin and eosin staining; IHC: immunohistochemical staining.

A total of 43 mice were randomly divided into three groups: healthy control mice (C group, n = 10); HCC mice (M group, n = 10); and nsPEF-treated HCC mice (N group, n = 23). Serum samples were collected from 42 mice (excluding one sample hemolysis), and fecal samples were completely collected from all 43 mice. The N group received nsPEF ablation (15 kV, 300 ns) 12 days after Hep1-6 cell implantation. The mice were euthanized after obtaining live images. Fecal samples were sequenced by 16S rRNA sequencing. Serum samples were analyzed by LC-MS. The weight and volume of the liver in the three groups were compared and analyzed (Supplementary Figure S1). Subsequently, the general tissue of the liver was stained with histopathological stains. The detailed study design is shown in Figure 1.

### 3.2 NsPEFs successfully ablated HCC in C57BL/6 mice

**FIGURE 2:**
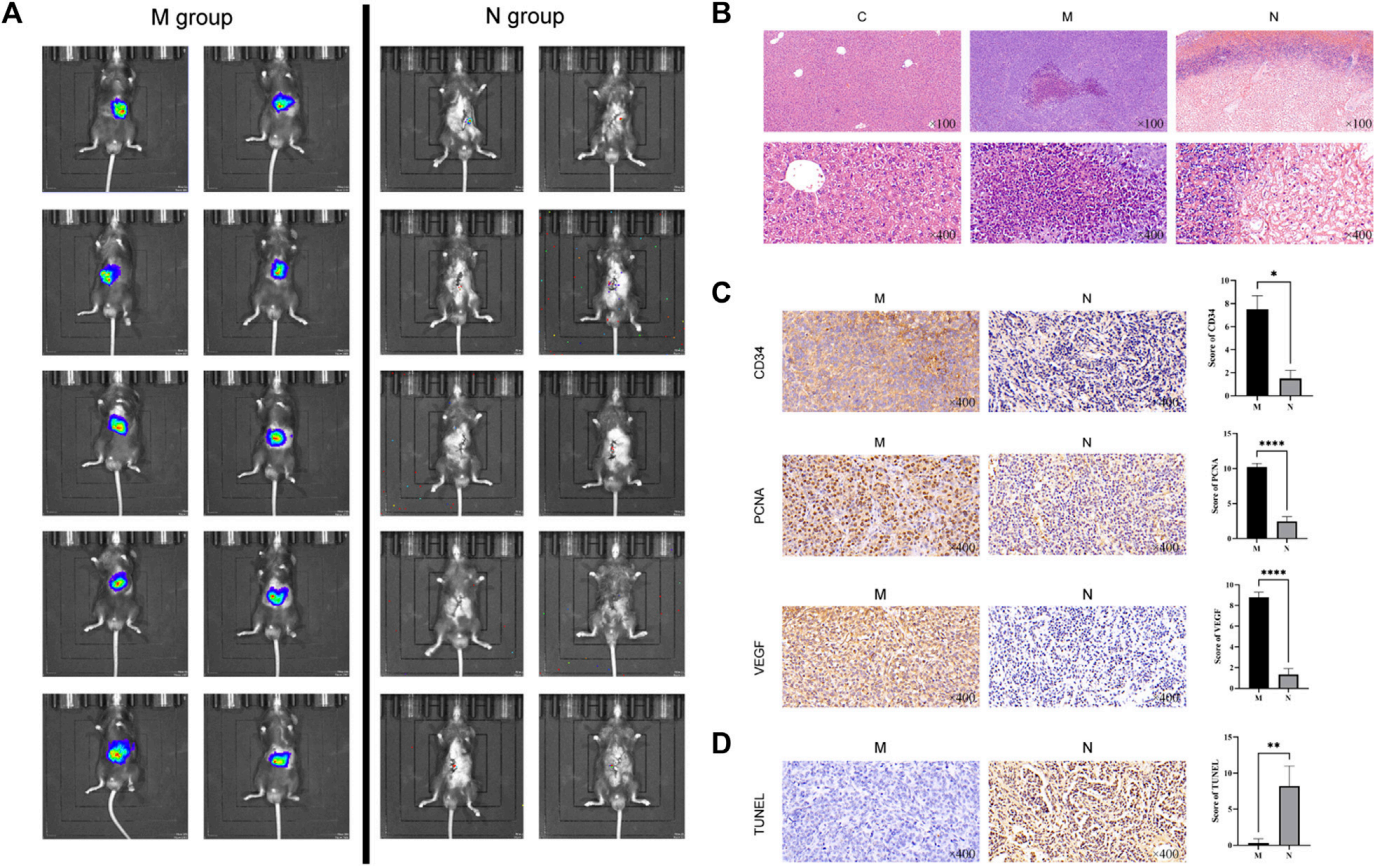
Live tumor image and the comparison of histopathological changes in healthy control mice (C group), HCC mice (M group), and nsPEF-treated HCC mice (N group). **(A)** Live tumor image in the M group and N group. **(B)** Comparison of normal liver tissue (C), tumor tissue (M), and nsPEF-treated tumor tissue (N) by HE staining, observed under a light microscope for 40 or 400 magnifications. Cell necrosis and apoptosis and lymphocytes and macrophages infiltrated around the ablated area. Original magnification × 100 and 400 ×. Apoptosis-related proteins including CD34, PCNA, and VEGF were determined by IHC **(C)**, and tumor apoptosis was detected by TUNEL assay **(D)**. IHC results were analyzed by GraphPad Prism 9. Original magnification ×400. *p < 0.05, **p < 0.01, and ****p < 0.0001.

On the third day after nsPEF ablation, the live image of the mice in the N group showed that the tumor volume decreased significantly and the metabolic activity slowed down compared with that in the M group (Figure 2A). Using hematoxylin and eosin (HE) staining, we observed morphological changes in HCC tissue (Figure 2B). By immunohistochemical (IHC) staining, we examined the ablation effect of nsPEFs (Figure 2C). The results of apoptosis staining are shown in Figure 2D. As shown in Figure 2B, compared with the HCC group, the tumor tissue of the N group showed significant cell necrosis and apoptosis, and lymphocytes and macrophages infiltrated around the ablated area.

CD34 is a highly differentiated glycosylated transmembrane glycoprotein selectively expressed on human and other mammalian stem/progenitor cell surfaces (Cui et al., 2018). It is also a marker of endothelial differentiation. CD34 is one of the specific markers of angiogenic tumors, and the positive results can be used to evaluate vascular invasion. CD34 staining was localized in the cytoplasm and showed uniform brownish-yellow particles. The microvessels of CD34-positive expression in HCC are long or branched, and the lumen is narrow and widely distributed. Compared with the obvious CD34-positive results in the M group, CD34 was almost unexpressed in the N group (*p* = 0.0251) (Figure 2C).

Proliferating cell nuclear antigen (PCNA) is closely related to DNA synthesis and plays an indispensable role in cell proliferation, which can be used as an indicator to evaluate the status of tumor tissue proliferation. PCNA existed in the nucleus, and the nuclei were uniformly stained when IHC staining was positive (*p* < 0.0001)**.** In the process of tumor development, the generation of blood vessels is one of the essential conditions.

Vascular endothelial growth factor (VEGF) is a highly specific growth factor promoting vascular endothelial cells, which can promote vascular permeability, extracellular matrix degeneration, vascular endothelial cell migration, proliferation, and vascular formation. VEGF is mainly expressed in the cytoplasm of glandular cells and part of matrix vascular endothelial cells. Brownish-yellow particles appear in the cytoplasm when positive. Compared with the strong positive in the M group, tumor tissue angiogenesis was significantly reduced after nsPEF treatment (*p* < 0.0001)**.**

The assay of TdT-dUTP terminal nick-end labeling (TUNEL) can detect the breakdown of nuclear DNA during cell apoptosis. Because normal or proliferating cells do not have DNA breaks, they will not be stained. In the N group, the number of TUNEL-positive cells significantly increased, which indicates that extensive apoptosis occurs after nsPEF ablation in local tumor tissue (*p* = 0.0085) (Figure 2D).

Our experimental results show that nsPEFs functioned as an effective tool by promoting cancer cell apoptosis, inhibiting tumor tissue angiogenesis, and changing the microenvironment for tumor growth *in vivo*.

### 3.3 Difference in the gut microbiome among the three groups

First, the rarefaction curve and rank abundance curve (Supplementary Figure S2) show that our sequencing data of the sample are reasonable. The analysis results can effectively reflect the microbial information of most samples.

**FIGURE 3:**
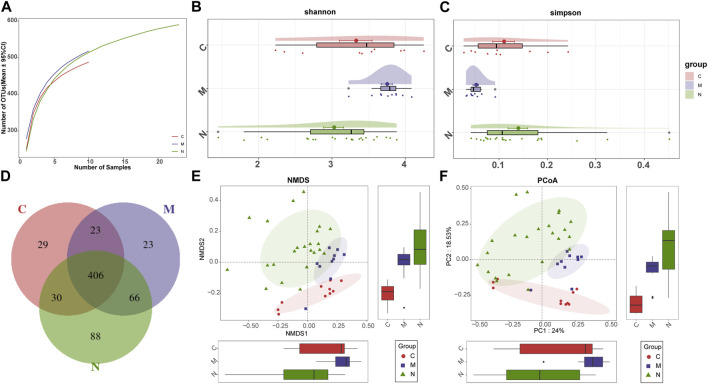
Gut microbiome diversity in the C group (n = 10), M group (n = 10), and N group (n = 23). **(A)** Specaccum (species accumulation curves) indicated the sufficient sampling size. Compared with the C group and N group, gut microbiome diversity, calculated by the Shannon index **(B)** and Simpson index **(C)**, was significantly increased in the M group (*p* < 0.001. Kruskal–Wallis test). **(D)** 406 of the 665 OTUs were shared among the three groups as shown by the Venn diagram. The significant difference was found among the C group, M group, and N group by **(E)** NMDS analysis and **(F)** PCoA, which indicated that the composition of the overall oral microbiota of AIH and HCs was different. C group, healthy mice; N group, nsPEF-treated mice; and M group, HCC mice. OTUs, operational taxonomic units; NMDS, non-metric multidimensional scaling; and PCoA, principal coordinate analysis.

As shown in the species accumulation curves (Figure 3A), the trend gradually flattens out from the initial sharp rise, indicating that the sample size is sufficient for analysis. The diversity of the gut microbiome was calculated by the Shannon index (Figure 3B) and Simpson index (Figure 3C). Compared with the N group, the diversity of the gut microbiome in the M group was increased significantly. The performance of the N group was also significantly different from that of the C group (*p* < 0.001; Kruskal–Wallis test). In addition, the Venn diagram (Figure 3D) shows overlapping relationships among groups displaying the same result. As shown, 406 of 665 OTUs were shared among the three groups. Notably, 88 of 665 OTUs were unique for the mice after nsPEFs.

Beta diversity was counted through non-metric multidimensional scaling (NMDS) analysis and principal coordinate analysis (PCoA), to deduce the microbiome space among the three groups. NMDS analysis of Bray–Curtis (Figure 3E) and PCoA of Bray–Curtis PC1-2 (Figure 3F) demonstrated that the samples of the C group and the other two groups were separated in the direction of the NMDS2, PC2, and PC2 axes, indicating that the overall fecal microbial composition was different between healthy mice and others. Furthermore, the samples of the M group and N group were observably separated in the direction of the NMDS1, PC1, and PC1 axes. The difference between these two groups can be confirmed through the aforementioned analysis.

### 3.4 Composition and comparison of the gut microbiome among the three groups

Concerning the composition of the gut microbiome among healthy mice, HCC mice, and nsPEF mice, according to the explanatory note of OTUs, the relative abundance of each sample was computed and plotted at each taxonomic level.

**FIGURE 4:**
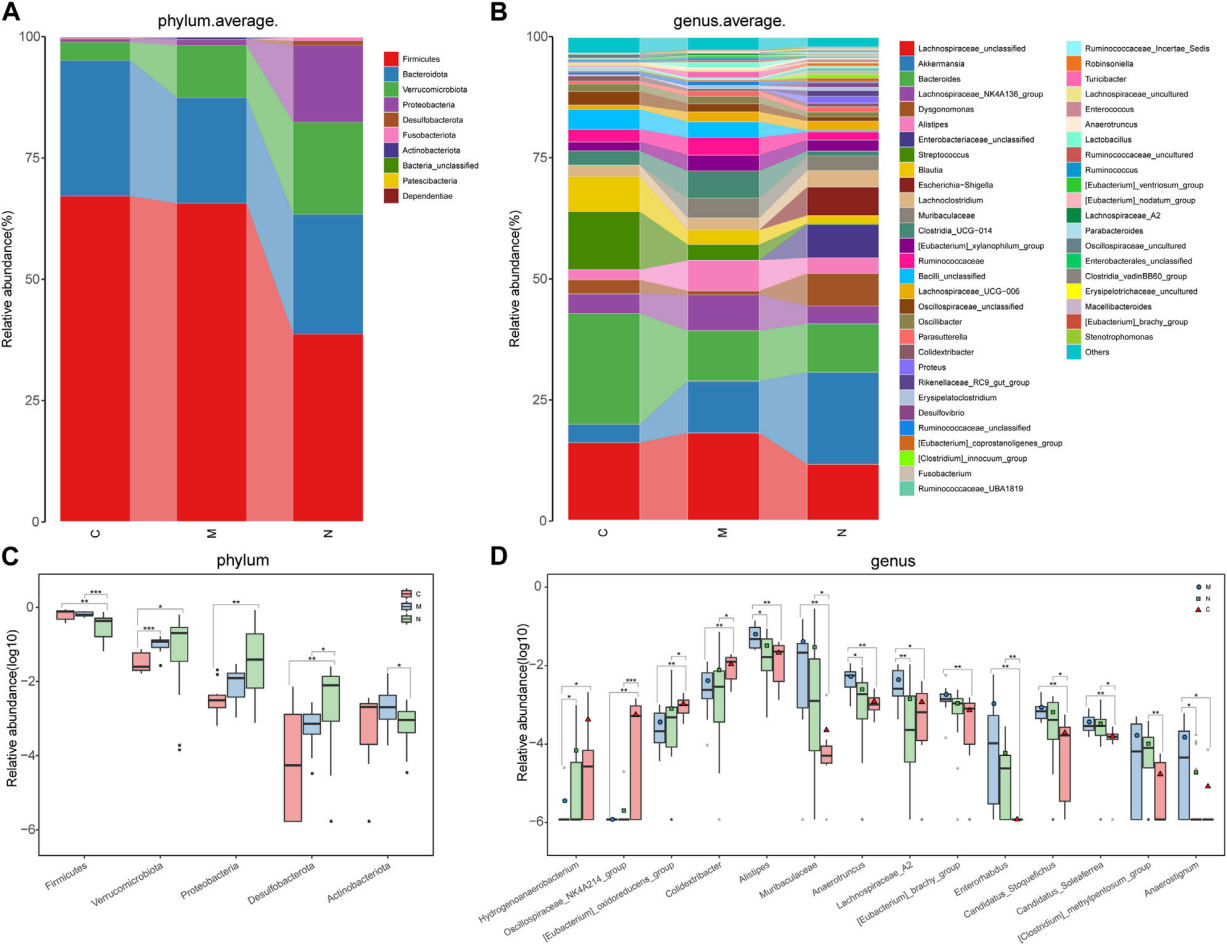
Composition and comparison of the gut microbiome in the C group (n = 10), M group (n = 10), and N group (n = 23). **(A)** The phylum-level and **(B)**genus-level composition diagrams showed the composition characteristics of the three groups of the gut microbiome. The differences in the relative abundance of key bacteria in the three groups were compared at the **(C)** phylum level and **(D)** genus level. The relative abundance of each bacterium was represented by the mean ± SE. We used the Kruskal–Wallis test to evaluate whether the difference of relative abundance was significant (*p < 0.05; **p < 0.01, and ***p < 0.001). C group, healthy mice; N group, nsPEF-treated mice; and M group, HCC mice.

The average proportion of *Firmicutes*, *Bacteroidota*, *Verrucomicrobiota*, and *Proteobacteria* in the three groups was up to 90% at the phylum level (Figure 4A). Delightedly, notable divergences of these four main phyla were detected among the three groups. Similarly, 18 genera, including *Lachnospiraceae_unclassified*, *Akkermansia, Bacteroides, Lachnospiraceae_NK4A136_group, Dysgonomonas,* and *Alistipes,* at the genus level, accounted for an average of more than 80% in the three groups (Figure 4B). At the phylum and genus levels, the microbial composition of the N group was distinct from that of the C group and M group.

Sequentially, the comparative analysis was carried out in the C group (n = 10), M group (n = 10), and N group (n = 23) at each taxonomic level. At the phylum level, the abundance of three phyla, namely, *Verrucomicrobiota*, *Proteobacteria*, and *Desulfobacterota,* increased gradually among the three groups (*p* < 0.05). In contrast, the abundance of *Firmicutes* and *Actinobacteria* was markedly decreased in the N group compared with the C group and M group (*p* < 0.05) (Figure 4C).

As observed at the genus level, four genera, namely, *Hydrogenoanaerobacterium*, *Oscillospiraceae_NK4A214_group,* (*Eubacterium*)*_oxidoreducens_group*, and *Colidextribacter*, increased in the following order: M group, N group, and C group (Figure 4D). Inversely, eight genera, including *Alistipes, Muribaculaceae, Anaerotruncus,* and (*Eubacterium*)*_brachy_group,* were gradually decreased in the aforementioned sequence. In particular, the abundance of *Lachnospiraceae_A2* is the highest in the M group and the lowest in the N group.

The boxplot at the phylum and genus levels indicated that there was a significant difference in the distribution of the gut microbiome among the three groups. Similar results were also concluded at the class level, order level, and family level (Supplementary Figure S3) (*p* < 0.05).

### 3.5 Operational taxonomic unit (OTU) clustering and taxonomic analysis

**FIGURE 5:**
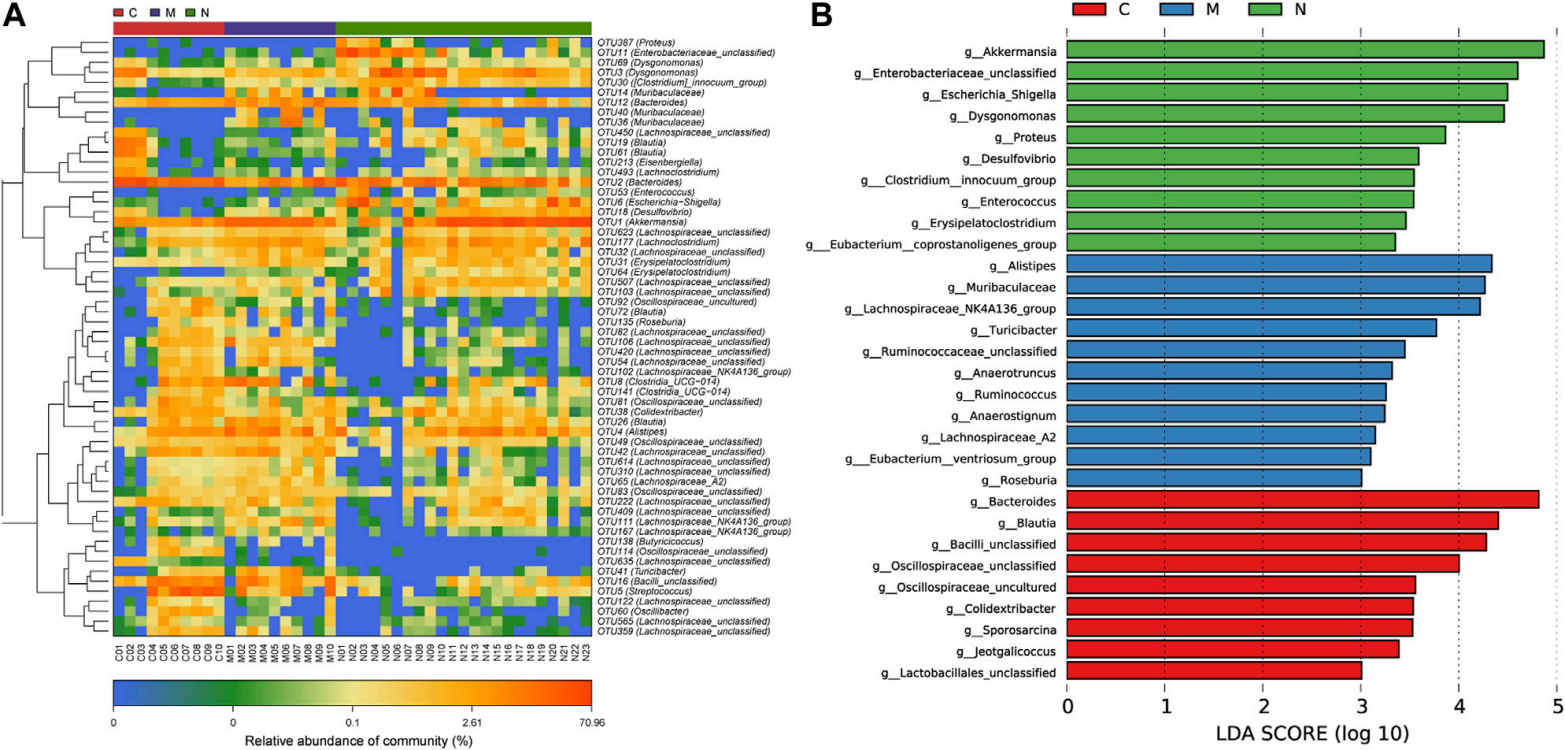
Heatmap and LDA based on OTU characterization of the microbiome among the C group (n = 10), M group (n = 10), and N group (n = 23). **(A)** The relative abundance for differential OTUs is shown on the right. The relative abundance of each OTU was used to plot the heatmap (blue, low abundance; red, high abundance). **(B)** Histogram of LDA scores calculated for selected taxa showing significant difference in microbe type and abundance among the C group (red), M group (blue), and N group (green). The score of LDA was positively correlated with the importance of the microbial marker. OTUs, operational taxonomic units; C group, healthy mice; N group, nsPEF-treated mice; and M group, HCC mice; and LDA, linear discriminant analysis.

To demonstrate the divergence more distinctly, we use the microbial community heatmap to show the distinction among the three groups (Figure 5A). The closer the color is to blue, the lower the relative abundance of each OTU. On the contrary, the closer the color is to red, the higher the relative abundance of each OTU.

According to the LEfSe and LDA score (Figure 5B), based on OTUs’ characterizing microbiota among the C group, M group, and N group, 10 genera were certified to be differential species for nsPEFs. Meanwhile, nine genera were considered to be dominant in the C group, 11 genera were considered to be dominant in the M group, and the difference among the three groups was of high significance.

### 3.6 Differences in serum metabolite composition among the three groups

**FIGURE 6:**
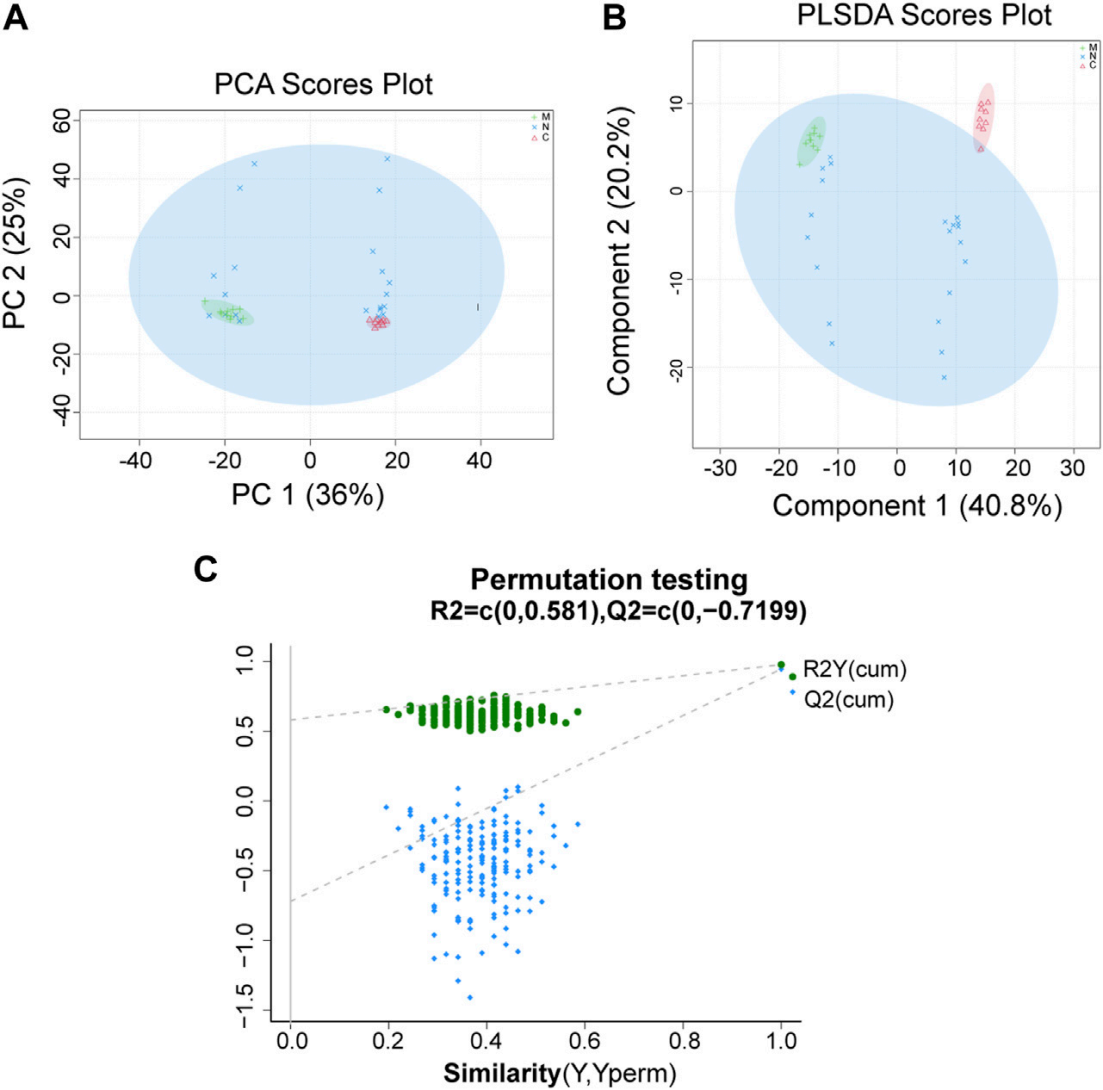
PCA and PLS-DA show the differences of the serum metabolite among the three groups. A significant difference was found among the C group, M group, and N group by **(A)** PCA and **(B)** PLS-DA, which indicated that the composition of the overall serum metabolite was different among the three groups. **(C)** Permutation testing shows that the model fits well. C group, healthy mice; M group, HCC mice; N group, nsPEF-treated mice. PCA, principal component analysis; PLS-DA, partial least squares-discrimination analysis.

For data preprocessing and annotation, we import the original data to the Progenesis QI, which is a proprietary metabolomics processing software. The serum samples of healthy mice (C group, n = 10), HCC mice (M group, n = 10), and nsPEF-treated mice (N group, n = 22) were collected and detected by LC-MS non-targeted metabonomics. A total of 897 metabolites were found, of which 279 were significantly different among the three groups (Supplementary Table S1). The distance between different observed samples can be intuitively observed in the principal component analysis (PCA) score plot, based on which the difference or similarity of the samples was analyzed. The principle of partial least squares-discriminant analysis (PLS-DA) is similar to that of PCA, and the PLS-DA model may be more effective for samples with less significant differences between groups. As shown in Figure 6A and Figure 6B, the characteristics of the metabolites among the three groups were significantly different. The permutation test (Figure 6C) used to evaluate the accuracy of the PLS-DA score plot shows that all Q2 values on the left are lower than the original point on the right, from which we can conclude that the model fitting effect is good.

### 3.7 Expression analysis of differential metabolites

**FIGURE 7:**
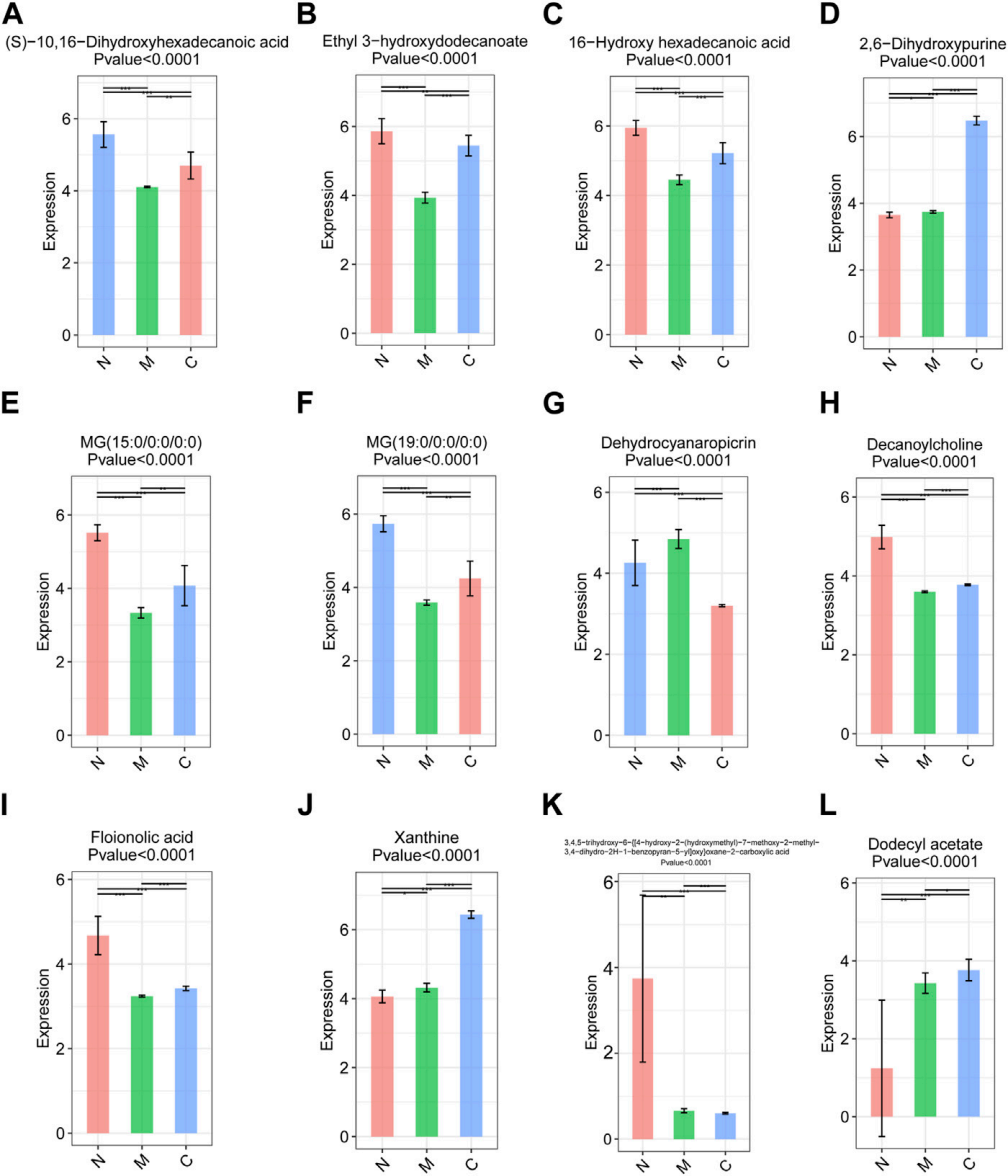
Top 12 differential metabolites among the C group (n = 10), M group (n = 10), and N group (n = 23). Metabolites satisfying VIP >2 and *p* < 0.05 were screened out, and there were differences among the three groups. VIP, variable importance. C group, healthy mice; M group, HCC mice; N group, nsPEF-treated mice.

The original data were imported into the metabonomics processing software Progenesis QI for preprocessing and analyzing. Through one-way analysis of variance combined with multivariate analysis, we identified 16 differential metabolites with significant differences among the three groups with strict screening conditions (both VIP >2 and *p* < 0.05). Among them, 12 metabolites, including (S)-10, 16-dihydroxyhexadecanoic acid, xanthine, 2,6-dihydroxypurine, and ethyl 3-hydroxydodecanoate, were significantly different between the C and M, C and M, and N and M groups. It was worth noting that the expression of (S)-10, 16-dihydroxyhexadecanoic acid (Figure 7A), ethyl 3-hydroxydodecanoate (Figure 7B), 16-hydroxyhexadecanoic acid (Figure 7C), MG (15:0/0:0/0:0) (Figure 7E), MG (19:0/0:0/0:0) (Figure 7F), decanoylcholine (Figure 7H), and phloionolic acid (Figure 7I) was the highest in the N group and the lowest in the M group. The expression of these metabolites in the C group was between N and M, indicating that these metabolites may be involved in the process of returning to normal in HCC mice after nsPEFs. Three metabolites, namely, 2,6-dihydroxypurine (Figure 7D), xanthine (Figure 7J), and dodecyl acetate (Figure 7L), have similar characteristics that were highest in the C group and lowest in the N group. Dehydrocyanaropicrin (Figure 7G) was significantly upregulated in the M group, while 3,4,5-trihydroxy-6-{[4-hydroxy-2-(hydroxymethyl)-7-methoxy-2-methyl-3,4-dihydr o-2H-1-benzopyran-5-yl]oxy}oxane-2-carboxylic acid (Figure 7K) was the most expressed in the N group.

### 3.8 Correlation analysis between serum metabolism and gut microbiome

Finally, we used Spearman’s rank correlation analysis to clarify the correlation between the gut microbiome and serum metabolism of the three groups and to find out the synergistic or opposite changes in the gut microbiome structure and serum metabolism in three different physiological or pathological states.

**FIGURE 8:**
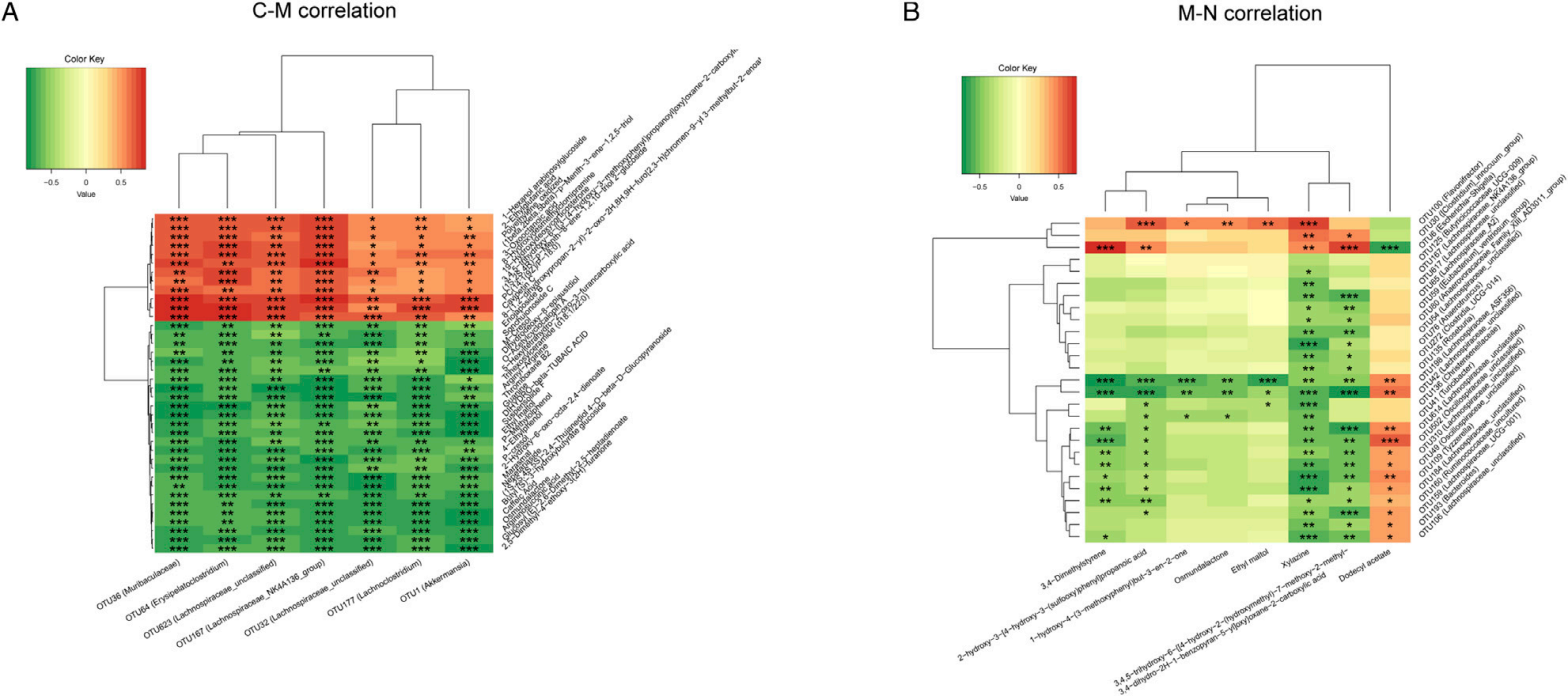
Heatmap of the correlation analysis between the gut microbiome and serum metabolism. **(A)** The correlation between the gut microbiome and serum metabolites was analyzed in the C group and M group. **(B)** The correlation between the gut microbiome and serum metabolites was analyzed in the M group and N group.

Figure 8A shows seven OTUs and 38 differential metabolites associated between the C group and M group. Seven kinds of OTUs, including OTU1 (*Akkermansia*) and OTU36 (Muribaculaceae), were positively correlated with 12 metabolites including 2-ethyl glutaric acid and 3-oxooctanoic acid and negatively correlated with 26 metabolites such as 4-ethylphenol and ethylphenol. The correlation analysis of OTUs and differential metabolites between the M group and N group is shown in Figure 8B. Dodecyl acetate was negatively correlated with OTU6 (*Escherichia Shigella*), OTU30 (*Clostridium_innocuum_group*), and OTU100 (*Flavonifractor*), but positively correlated with 24 OTUs, including OTU41 (*Turicibacter*) and OTU76 (*Anaerotruncus*).

Similar results were found in the C group and N group (Supplementary Figure S4).

## 4 Discussion

HCC has high morbidity and mortality, frequent recurrence and metastasis, and poor prognosis (Forner et al., 2018). In this study, the effectiveness of nsPEF ablation in HCC mice was confirmed from three aspects: pathological characteristics, gut microbiome composition, and serum metabolite diversity. We identified the differences in the fecal microbiome and serum metabonomics among normal mice, HCC mice, and nsPEF-treated mice. At the histopathological level, we identified the differences between groups by HE and IHC staining.

At different stages of progression from chronic liver disease to HCC, microflora disorders were observed, that is, changes in the composition of the gut microbiome. The gut microbiome can inhibit immune surveillance and promote tumor growth through the transformation of primary bile acid into secondary bile acid. The importance of the gut microbiome in regulating systemic immunity has been widely recognized. Previous studies have shown (Yin et al., 2016; Qian et al., 2020) that nsPEF, as a local treatment, can stimulate a systemic immune activation effect and prevent HCC from metastasizing in the liver and developing in distant areas. There have been microecological studies suggesting that the microecology and metabolism of the digestive tract play an important role in this process (Dong et al., 2021).

Angiogenesis plays an important role in rapid tumor growth and proliferation in surrounding tissues. As a highly glycosylated transmembrane glycoprotein, CD34 protein can be expressed on the surface of human hematopoietic stem cells. It is a commonly used vascular endothelial marker in immunohistochemical staining of tissues. Amarapurkar et al. reported immunohistochemistry staining of CD34 in normal liver, cirrhosis, HCC, and metastatic carcinoma of the liver. The expression of CD34 increased gradually with cirrhosis, carcinogenesis, and metastasis (Amarapurkar et al., 2008). None of the normal tissues showed positive staining on the CD34 protein, which is the same as our results. In the N group, the expression of CD34 in tumor tissues was significantly lower than that in HCC tissues after nsPEF treatment, which indicated that nsPEFs had the effect of anti-angiogenesis in tumor therapy. VEGF is expressed on the surface of vascular endothelial cells, as a ligand of the tyrosine kinase receptor, and mediates neovascularization by inducing endothelial cell proliferation, migration, and vascular permeability (Vanderborght et al., 2020). It has long been reported that upregulation of VEGF is associated with the invasiveness and poor prognosis of HCC (Zhang et al., 2006). Angiogenesis is one of the markers of malignant tumor formation. Based on this theory, drugs such as bevacizumab, sorafenib, and pazopanib play an anticancerous effect by inhibiting angiogenesis in different pathways (Itatani et al., 2018). The anti-angiogenic effect of nsPEFs has been reported previously. Ren et al. observed vascular phagocytosis and cell atrophy in tumor tissues after nsPEF ablation by transmission electron microscopy and IHC staining. Consistent with our findings, the expression of angiogenesis marker proteins, VEGF and CD34, was downregulated in tissues after treatment (Ren et al., 2013).

PCNA exists in normal proliferating cells and tumor cells, and IHC staining can show significant positive results when tumor tissues are formed. Feng et al. reported that PCNA enhances hepatitis B virus (HBV) replication through covalently closed circular DNA (cccDNA) interaction with HBV, thus accelerating the occurrence of HCC (Feng et al., 2019). A clinical study has reported that there is a negative correlation between the enrichment of CD34 and PCNA. In AFP-negative HCC patients, the expression of CD34 is high and that of PCNA is low. In our study, CD34 and PCNA were significantly upregulated in HCC mice, but significantly downregulated after treatment with nsPEFs. The limitation in our study is that we did not detect the level of serum AFP in mice (Cui et al., 2018). TUNEL, contrary to PCNA, is an indicator of the degree of apoptosis. PCNA showed positive results in the M group and was significantly downregulated in the N group, while TUNEL was not expressed in the M group, but significantly up-regulated in the N group. This is strong evidence that nsPEFs inhibit the proliferation of tumor cells.

Our study is the first to report the characteristics of the gut microbiome and serum metabonomics in mice after nsPEF ablation. A total of 21 normal mice, 10 HCC model mice, and 10 nsPEF-treated mice underwent 16S rRNA gene sequencing. The results displayed that the total microbial composition was significantly different among the three groups. Seven genera, including *Bacteroides, Streptococcus,* and *Blautia*, were enriched in the C group, while *Akkermansia, Lachnospiraceae_NK4A136_group, Alistipes,* and *Clostridia_UCG-014* were enriched in the M group. As for the N group, the abundance of *Dysgonomonas, Enterobacteriaceae_unclassified, Escherichia Shigella,* and *Proteus* was significantly higher than that of the other two groups. The NMDS analysis and PCoA confirmed a significant divergence among the C, M, and N groups in terms of gut microbiome community composition.

The findings of this study confirm previous reports that nsPEFs play an antitumor effect by inducing tumor cell apoptosis and inhibiting neovascularization (Ren et al., 2013; Miao et al., 2015; Rossi et al., 2019). Our innovation is to confirm the effectiveness of nsPEFs as a new generation of minimally invasive ablation tools for HCC through gut microbiome and serum metabonomics analysis. In the study by Dong et al., pigs were treated with nsPEFs, and the effects of nsPEFs on serum metabolism and gut microbiome were observed by multiomics analysis (Dong et al., 2021). However, their research focused on the metabolic changes associated with liver injury and the changes in microflora pre- and post-treatment, and there was no animal model of HCC.

The stable gut microbiome provides internal environmental support for the normal metabolism of the host. When the normal gut microbiome is changed and pathogenic bacteria invade, it will lead to a variety of diseases. In our study, compared with healthy mice, the gut microbiota diversity of HCC mice increased significantly. Eight genera including *Alistipes, Muribaculaceae, Anaerotruncus,* and *Lachnospiraceae_A2* were predominant in the M group, while they decreased in the N group and were lowest in the C group.

*Alistipes* belongs to the *Bacteroidetes* phylum. *Alistipes* has been isolated from the feces of patients with appendicitis and rectal abscess (Parker et al., 2020). In the gut microbiome of patients with major depressive disorder, the abundance of *Alistipes* was detected to increase significantly. The abundance of Muribaculaceae was detected in patients with *cholangiocarcinoma* (Jiang et al., 2015), whereas the abundance of *Muribaculaceae* was detected in patients with cholangiocarcinoma (Zhang et al., 2021). *Anaerotruncus* has also been reported to play a role in the obesity process in mice (Kong et al., 2019). By referring to the literature, we found that the gut microbiome also changed in the process of CRC (Yang et al., 2022), diabetes (Zhuang et al., 2021), aging (Ma et al., 2020), and other diseases. Therefore, we speculate that the increase of these harmful bacteria in the gut microbiome may be involved in the progression of HCC, and the gut microbiome will gradually transition to normal after effective treatment with nsPEFs.

The gut microbiota plays an indispensable role in the occurrence and development of many diseases. In previous studies, we have reported changes in the gut microbiota of normal people and cirrhotic and HCC patients (Ren et al., 2019). Through cross-regional verification, the candidate bacteria that may cause the occurrence and development of HCC were found. The gut microbiota is also used as a convenient non-invasive diagnostic tool in other common liver diseases such as primary biliary cholangitis (PBC) (Tang et al., 2018), autoimmune hepatitis (AIH) (Lou et al., 2020), and NAFLD (Caussy et al., 2019), and the prognosis model is established by the gut microbiome.

Because of its convenient and non-invasive characteristics, serum metabonomics is increasingly used in the diagnosis of diseases and the establishment of prognostic models. For example, in NAFLD (Lewinska et al., 2021), liver failure (Yu et al., 2021), PBC (Bell et al., 2015), and other diseases, through the metabonomics analysis of the serum of normal and diseased people, we can well-distinguish between normal and diseased people. Metabonomics is also often combined with gut microbiome analysis to establish multi-group diagnostic models (Gao et al., 2019; Gong et al., 2020; Lu et al., 2021). In our study, through serum metabonomics and gut microbiome, we found that HCC mice had flora and metabolic disorders in the process of disease progression, and we also confirmed the effectiveness of nsPEFs in the treatment of HCC in animal models.

Some bacterial abundance was positively correlated with the production of serum metabolites, while some showed an obvious negative correlation. The correlation analysis between metabolism and microorganisms provides a new perspective for the multiomics study of nsPEFs. In short, under the effect of nsPEFs, the flora disorder was modified, and the intestinal microorganisms played a role by interfering with the metabolic pathway of serum.

As an electric field-dependent ablation technique, the primary target of nsPEFs is cellular DNA (Chen et al., 2012). Under the stimulation of a high-intensity electric field and ultrashort pulse, the double helix structure of DNA is irreversibly destroyed, which eventually leads to apoptosis after a series of cascade reactions. This process is accompanied by the rupture of the cell membrane and plasma membrane. In a recent report, clinical trials were conducted using nsPEFs in HCC patients (Xu et al., 2022).

The limitation of our study is that there is no detection of serum biochemical indexes in HCC and nsPEF-treated mice. As a substantial organ of the human body, the liver participates in a variety of metabolic reactions, and the destruction of hepatocytes is often accompanied by the abnormality of serum biochemical indexes. It has been reported that liver function will be enhanced temporarily within a few days after nsPEF ablation and return to normal after 7 days (Dong et al., 2021). For the HCC mice, whether it will also cause transient liver injury after nsPEF ablation and the duration of liver injury remain to be explored. Furthermore, we have found that there is a significant correlation between serum metabolites and gut microbiome in mice after nsPEF ablation. We speculate that intestinal microorganisms may affect the health of the host by interfering with some metabolites in the metabolic pathway, but the real causal relationship between the two requires further study.

The liver has two sets of blood supply systems, the portal vein and hepatic artery. The blood vessels are complex and diverse. The growth of HCC is inseparable from blood supply. Traditional thermal ablation methods such as radiofrequency ablation (RFA) and microwave ablation may cause thermal damage to the vascular and bile duct system of the adjacent lesions, resulting in complications. At the same time, blood vessels take away the heat from the ablation area, which will lead to incomplete ablation, which is one of the reasons for recurrence after RFA. The nsPEFs do not damage blood vessels and bile ducts and do not produce the heat-sink effect. They have a good ablation effect on high-risk tumors, such as, besides the bile duct, the blood vessel, the first hepatic portal area, and the second hepatic portal area. Therefore, it is of great significance to explore the serum metabonomics and gut microbiome of HCC mice after nsPEF ablation, which is of great significance for the microecological changes caused by nanosecond pulse and its possible mechanism.

In conclusion, as a new minimally invasive tumor ablation method, nsPEFs have a great application prospect. This study confirmed the effectiveness of nsPEFs in HCC mice and presented the characteristics of the gut microbiome and serum metabonomics in nsPEF-treated HCC mice for the first time. The local ablation of nanosecond pulses causes systematic effects. The nanosecond pulse is expected to break the immunosuppressive microenvironment and reshape the intestinal microecology in the digestive tract. This study provides a new perspective for intestinal microecology to activate the immune response and participate in immune reconstruction in nanosecond pulse, suggesting that the combination of pulsed electric field and microecology may be a new strategy for comprehensive treatment of HCC.

## Data Availability

The datasets presented in this study can be found in online repositories. The names of the repository/repositories and accession number(s) can be found in the article/Supplementary Material.

## References

[B1] Amarapurkar A. D. Vibhav Kim V. (2008). Angiogenesis in liver cirrhosis and hepatocellular carcinoma. Indian J. Pathol. Microbiol. 51 (3), 323–328. 10.4103/0377-4929.42504 18723951

[B2] Bell L. N. Wulff J. Comerford M. Vuppalanchi R. Chalasani N. (2015). Serum metabolic signatures of primary biliary cirrhosis and primary sclerosing cholangitis. Liver Int. 35 (1), 263–274. 10.1111/liv.12680 25181933 PMC4293304

[B3] Breton M. Mir L. M. (2012). Microsecond and nanosecond electric pulses in cancer treatments. Bioelectromagnetics 33 (2), 106–123. 10.1002/bem.20692 21812011

[B4] Cao W. Chen H. D. Yu Y. W. Li N. Chen W. Q. (2021). Changing profiles of cancer burden worldwide and in China: A secondary analysis of the global cancer statistics 2020. Chin. Med. J. Engl. 134 (7), 783–791. 10.1097/CM9.0000000000001474 33734139 PMC8104205

[B5] Caussy C. Tripathi A. Humphrey G. Bassirian S. Singh S. Faulkner C. (2019). A gut microbiome signature for cirrhosis due to nonalcoholic fatty liver disease. Nat. Commun. 10 (1), 1406. 10.1038/s41467-019-09455-9 30926798 PMC6440960

[B6] Chen X. Zhuang J. Kolb J. F. Schoenbach K. H. Beebe S. J. (2012). Long term survival of mice with hepatocellular carcinoma after pulse power ablation with nanosecond pulsed electric fields. Technol. Cancer Res. Treat. 11 (1), 83–93. 10.7785/tcrt.2012.500237 22181334

[B7] Chen X. Chen Y. Jiang J. Wu L. Yin S. Miao X. (2017). Nano-pulse stimulation (NPS) ablate tumors and inhibit lung metastasis on both canine spontaneous osteosarcoma and murine transplanted hepatocellular carcinoma with high metastatic potential. Oncotarget 8 (27), 44032–44039. 10.18632/oncotarget.17178 28476039 PMC5546459

[B8] Cui D. J. Wu Y. Wen D. H. (2018). CD34, PCNA and CK19 expressions in AFP- hepatocellular carcinoma. Eur. Rev. Med. Pharmacol. Sci. 22 (16), 5200–5205. 10.26355/eurrev_201808_15717 30178842

[B9] Dong Y. Lu J. Wang T. Huang Z. Chen X. Ren Z. (2021). Multi-omics analysis reveals disturbance of nanosecond pulsed electric field in the serum metabolic spectrum and gut microbiota. Front. Microbiol. 12, 649091. 10.3389/fmicb.2021.649091 34276585 PMC8283677

[B10] Fang Y. Zhang C. Shi H. Wei W. Shang J. Zheng R. (2021). Characteristics of the gut microbiota and metabolism in patients with latent autoimmune diabetes in adults: A case-control study. Diabetes Care 44 (12), 2738–2746. 10.2337/dc20-2975 34620611 PMC8669532

[B11] Feng Q. Liang S. Jia H. Stadlmayr A. Tang L. Lan Z. (2015). Gut microbiome development along the colorectal adenoma-carcinoma sequence. Nat. Commun. 6, 6528. 10.1038/ncomms7528 25758642

[B12] Feng J. Yang G. Liu Y. Gao Y. Zhao M. Bu Y. (2019). LncRNA PCNAP1 modulates Hepatitis B virus replication and enhances tumor growth of liver cancer. Theranostics 9 (18), 5227–5245. 10.7150/thno.34273 31410212 PMC6691589

[B13] Ford W. E. Ren W. Blackmore P. F. Schoenbach K. H. Beebe S. J. (2010). Nanosecond pulsed electric fields stimulate apoptosis without release of pro-apoptotic factors from mitochondria in B16f10 melanoma. Arch. Biochem. Biophys. 497 (1-2), 82–89. 10.1016/j.abb.2010.03.008 20346344

[B14] Forner A. Reig M. Bruix J. (2018). Hepatocellular carcinoma. Lancet 391 (10127), 1301–1314. 10.1016/s0140-6736(18)30010-2 29307467

[B15] Gao F. Lv Y. W. Long J. Chen J. M. He J. M. Ruan X. Z. (2019). Butyrate improves the metabolic disorder and gut microbiome dysbiosis in mice induced by a high-fat diet. Front. Pharmacol. 10, 1040. 10.3389/fphar.2019.01040 31607907 PMC6761375

[B16] Gong H. Zhang S. Li Q. Zuo C. Gao X. Zheng B. (2020). Gut microbiota compositional profile and serum metabolic phenotype in patients with primary open-angle glaucoma. Exp. Eye Res. 191, 107921. 10.1016/j.exer.2020.107921 31917963

[B17] Guo S. Burcus N. I. Hornef J. Jing Y. Jiang C. Heller R. (2018). Nano-pulse stimulation for the treatment of pancreatic cancer and the changes in immune profile. Cancers (Basel) 10 (7), 217. 10.3390/cancers10070217 29954062 PMC6070875

[B18] Hall Z. Chiarugi D. Charidemou E. Leslie J. Scott E. Pellegrinet L. (2021). Lipid remodeling in hepatocyte proliferation and hepatocellular carcinoma. Hepatology 73 (3), 1028–1044. 10.1002/hep.31391 32460431

[B19] Henshaw J. Mossop B. Yuan F. (2008). Relaxin treatment of solid tumors: Effects on electric field-mediated gene delivery. Mol. Cancer Ther. 7 (8), 2566–2573. 10.1158/1535-7163.MCT-08-0435 18723501 PMC2596587

[B20] Huang D. Q. El-Serag H. B. Loomba R. (2021). Global epidemiology of NAFLD-related HCC: Trends, predictions, risk factors and prevention. Nat. Rev. Gastroenterol. Hepatol. 18 (4), 223–238. 10.1038/s41575-020-00381-6 33349658 PMC8016738

[B21] Itatani Y. Kawada K. Yamamoto T. Sakai Y. (2018). Resistance to anti-angiogenic therapy in cancer-alterations to anti-VEGF pathway. Int. J. Mol. Sci. 19 (4), 1232. 10.3390/ijms19041232 29670046 PMC5979390

[B22] Jiang H. Ling Z. Zhang Y. Mao H. Ma Z. Yin Y. (2015). Altered fecal microbiota composition in patients with major depressive disorder. Brain Behav. Immun. 48, 186–194. 10.1016/j.bbi.2015.03.016 25882912

[B23] Kielbik A. Szlasa W. Novickij V. Szewczyk A. Maciejewska M. Saczko J. (2021). Effects of high-frequency nanosecond pulses on prostate cancer cells. Sci. Rep. 11 (1), 15835. 10.1038/s41598-021-95180-7 34349171 PMC8339066

[B24] Kong C. Gao R. Yan X. Huang L. Qin H. (2019). Probiotics improve gut microbiota dysbiosis in obese mice fed a high-fat or high-sucrose diet. Nutrition 60, 175–184. 10.1016/j.nut.2018.10.002 30611080

[B25] Lewinska M. Santos-Laso A. Arretxe E. Alonso C. Zhuravleva E. Jimenez-Agüero R. (2021). The altered serum lipidome and its diagnostic potential for Non-Alcoholic Fatty Liver (NAFL)-associated hepatocellular carcinoma. EBioMedicine 73, 103661. 10.1016/j.ebiom.2021.103661 34740106 PMC8577325

[B26] Li C. Ke Q. Yao C. Mi Y. Liu H. Lv Y. (2018). Cell electrofusion based on nanosecond/microsecond pulsed electric fields. PLoS One 13 (5), e0197167. 10.1371/journal.pone.0197167 29795594 PMC5967737

[B27] Lou J. Jiang Y. Rao B. Li A. Ding S. Yan H. (2020). Fecal microbiomes distinguish patients with autoimmune hepatitis from healthy individuals. Front. Cell Infect. Microbiol. 10, 342. 10.3389/fcimb.2020.00342 32850468 PMC7416601

[B28] Lu L. Tang M. Li J. Xie Y. Li Y. Xie J. (2021). Gut microbiota and serum metabolic signatures of high-fat-induced bone loss in mice. Front. Cell Infect. Microbiol. 11, 788576. 10.3389/fcimb.2021.788576 35004355 PMC8727351

[B29] Ma J. Hong Y. Zheng N. Xie G. Lyu Y. Gu Y. (2020). Gut microbiota remodeling reverses aging-associated inflammation and dysregulation of systemic bile acid homeostasis in mice sex-specifically. Gut Microbes 11 (5), 1450–1474. 10.1080/19490976.2020.1763770 32515683 PMC7524276

[B30] McGlinchey A. J. Govaere O. Geng D. Ratziu V. Allison M. Bousier J. (2022). Metabolic signatures across the full spectrum of non-alcoholic fatty liver disease. JHEP Rep. 4 (5), 100477. 10.1016/j.jhepr.2022.100477 35434590 PMC9006858

[B31] Miao X. Yin S. Shao Z. Zhang Y. Chen X. (2015). Nanosecond pulsed electric field inhibits proliferation and induces apoptosis in human osteosarcoma. J. Orthop. Surg. Res. 10, 104. 10.1186/s13018-015-0247-z 26148858 PMC4496869

[B32] Nuccitelli R. (2019). Nano-pulse stimulation therapy for the treatment of skin lesions. Bioelectricity 1 (4), 235–239. 10.1089/bioe.2019.0027 34471826 PMC8370293

[B33] Pakhomov A. G. Kolb J. F. White J. A. Joshi R. P. Xiao S. Schoenbach K. H. (2007). Long-lasting plasma membrane permeabilization in mammalian cells by nanosecond pulsed electric field (nsPEF). Bioelectromagnetics 28 (8), 655–663. 10.1002/bem.20354 17654532

[B34] Parker B. J. Wearsch P. A. Veloo A. C. M. Rodriguez-Palacios A. (2020). The genus Alistipes: Gut bacteria with emerging implications to inflammation, cancer, and mental health. Front. Immunol. 11, 906. 10.3389/fimmu.2020.00906 32582143 PMC7296073

[B35] Qian J. Chen T. Wu Q. Zhou L. Zhou W. Wu L. (2020). Blocking exposed PD-L1 elicited by nanosecond pulsed electric field reverses dysfunction of CD8(+) T cells in liver cancer. Cancer Lett. 495, 1–11. 10.1016/j.canlet.2020.09.015 32949680

[B36] Ren Z. Chen X. Cui G. Yin S. Chen L. Jiang J. (2013). Nanosecond pulsed electric field inhibits cancer growth followed by alteration in expressions of NF-κB and Wnt/β-catenin signaling molecules. PLoS One 8 (9), e74322. 10.1371/journal.pone.0074322 24069295 PMC3775773

[B37] Ren Z. Li A. Jiang J. Zhou L. Yu Z. Lu H. (2019). Gut microbiome analysis as a tool towards targeted non-invasive biomarkers for early hepatocellular carcinoma. Gut 68 (6), 1014–1023. 10.1136/gutjnl-2017-315084 30045880 PMC6580753

[B38] Ren Z. Fan Y. Li A. Shen Q. Wu J. Ren L. (2020). Alterations of the human gut microbiome in chronic kidney disease. Adv. Sci. (Weinh) 7 (20), 2001936. 10.1002/advs.202001936 33101877 PMC7578882

[B39] Rossi A. Pakhomova O. N. Pakhomov A. G. Weygandt S. Bulysheva A. A. Murray L. E. (2019). Mechanisms and immunogenicity of nsPEF-induced cell death in B16F10 melanoma tumors. Sci. Rep. 9 (1), 431. 10.1038/s41598-018-36527-5 30674926 PMC6344591

[B40] Tang R. Wei Y. Li Y. Chen W. Chen H. Wang Q. (2018). Gut microbial profile is altered in primary biliary cholangitis and partially restored after UDCA therapy. Gut 67 (3), 534–541. 10.1136/gutjnl-2016-313332 28213609

[B41] Ussher J. R. Elmariah S. Gerszten R. E. Dyck J. R. (2016). The emerging role of metabolomics in the diagnosis and prognosis of cardiovascular disease. J. Am. Coll. Cardiol. 68 (25), 2850–2870. 10.1016/j.jacc.2016.09.972 28007146

[B42] Vanderborght B. Lefere S. Vlierberghe H. V. Devisscher L. (2020). The angiopoietin/tie2 pathway in hepatocellular carcinoma. Cells 9 (11), 2382. 10.3390/cells9112382 33143149 PMC7693961

[B43] Wang Q. Tan Y. Jiang T. Wang X. Li Q. Li Y. (2022). Metabolic reprogramming and its relationship to survival in hepatocellular carcinoma. Cells 11 (7), 1066. 10.3390/cells11071066 35406630 PMC8997969

[B44] Weiss R. H. Kim K. (2011). Metabolomics in the study of kidney diseases. Nat. Rev. Nephrol. 8 (1), 22–33. 10.1038/nrneph.2011.152 22025087

[B45] Xu X. Chen Y. Zhang R. Miao X. Chen X. (2018). Activation of anti-tumor immune response by ablation of HCC with nanosecond pulsed electric field. J. Clin. Transl. Hepatol. 6 (1), 85–88. 10.14218/JCTH.2017.00042 29577034 PMC5863003

[B46] Xu M. Xu D. Dong G. Ren Z. Zhang W. Aji T. (2022). The safety and efficacy of nanosecond pulsed electric field in patients with hepatocellular carcinoma: A prospective phase 1 clinical study protocol. Front. Oncol. 12, 869316. 10.3389/fonc.2022.869316 35912221 PMC9328750

[B47] Yang J. Wei H. Zhou Y. Szeto C. H. Li C. Lin Y. (2022). High-fat diet promotes colorectal tumorigenesis through modulating gut microbiota and metabolites. Gastroenterology 162 (1), 135–149.e2. 10.1053/j.gastro.2021.08.041 34461052

[B48] Yimingjiang M. Tuergan T. Chen X. Wen H. Shao Y. Zhang R. (2020). Comparative analysis of immunoactivation by nanosecond pulsed electric fields and PD-1 blockade in murine hepatocellular carcinoma. Anal. Cell Pathol. (Amst) 2020, 9582731. 10.1155/2020/9582731 32802733 PMC7416239

[B49] Yin S. Chen X. Xie H. Zhou L. Guo D. Xu Y. (2016). Nanosecond pulsed electric field (nsPEF) enhance cytotoxicity of cisplatin to hepatocellular cells by microdomain disruption on plasma membrane. Exp. Cell Res. 346 (2), 233–240. 10.1016/j.yexcr.2016.06.018 27375200

[B50] Yu M. Zhou C. Tian D. Jia H. M. Li Z. Q. Yang C. (2021). Molecular classification and clinical diagnosis of acute-on-chronic liver failure patients by serum metabolomics. J. Pharm. Biomed. Anal. 198, 114004. 10.1016/j.jpba.2021.114004 33721610

[B51] Zhang Z. L. Liu Z. S. Sun Q. (2006). Expression of angiopoietins, Tie2 and vascular endothelial growth factor in angiogenesis and progression of hepatocellular carcinoma. World J. Gastroenterol. 12 (26), 4241–4245. 10.3748/wjg.v12.i26.4241 16830384 PMC4087383

[B52] Zhang T. Zhang S. Jin C. Lin Z. Deng T. Xie X. (2021). A predictive model based on the gut microbiota improves the diagnostic effect in patients with cholangiocarcinoma. Front. Cell. Infect. Microbiol. 11, 751795. 10.3389/fcimb.2021.751795 34888258 PMC8650695

[B53] Zhuang P. Li H. Jia W. Shou Q. Zhu Y. Mao L. (2021). Eicosapentaenoic and docosahexaenoic acids attenuate hyperglycemia through the microbiome-gut-organs axis in db/db mice. Microbiome 9 (1), 185. 10.1186/s40168-021-01126-6 34507608 PMC8434703

