# Supplementary figures and images for: Nanosecond pulse effectively ablated hepatocellular carcinoma with alterations in the gut microbiome and serum metabolites
Source: Front Pharmacol. 2023 May 10;14:1163628. doi: 10.3389/fphar.2023.1163628 (PMC10205996; doi:10.3389/fphar.2023.1163628)

## family

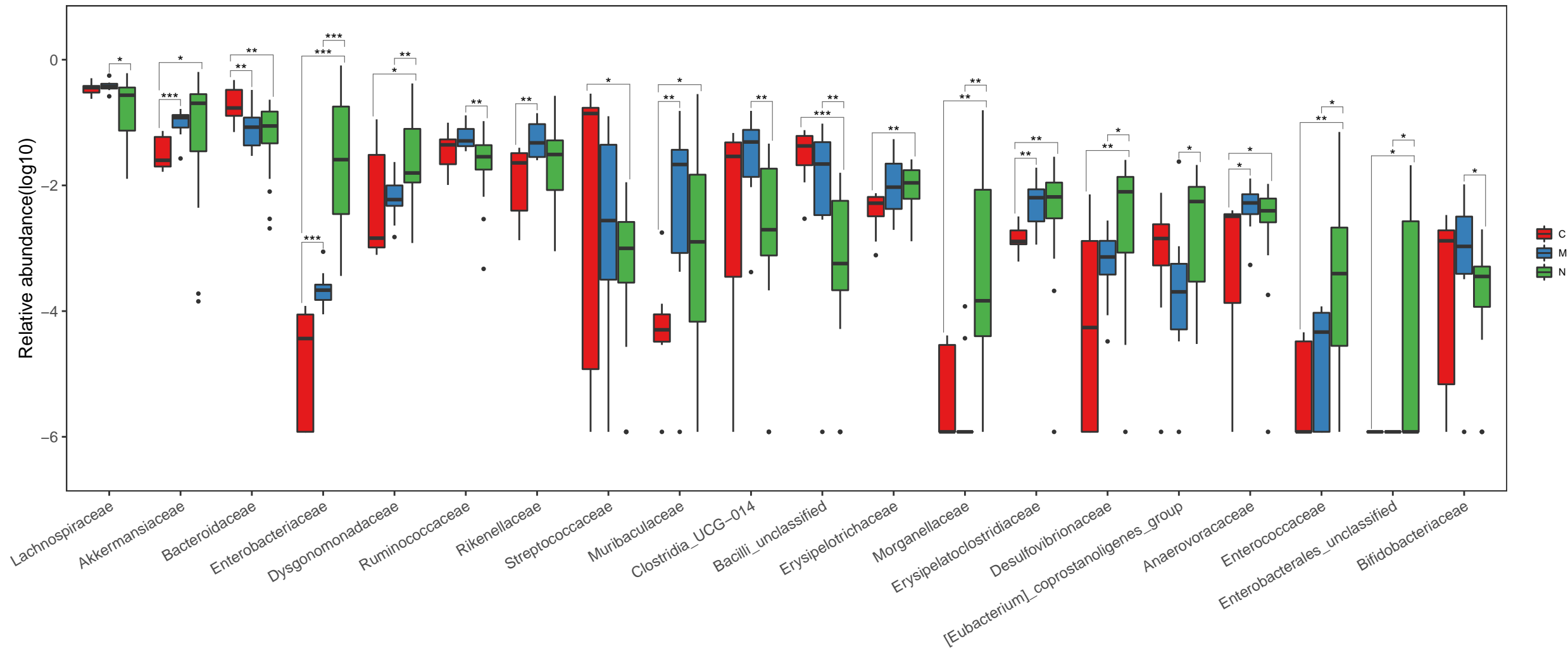

Supplement: Supplementary file 1 [file DataSheet2.PDF]

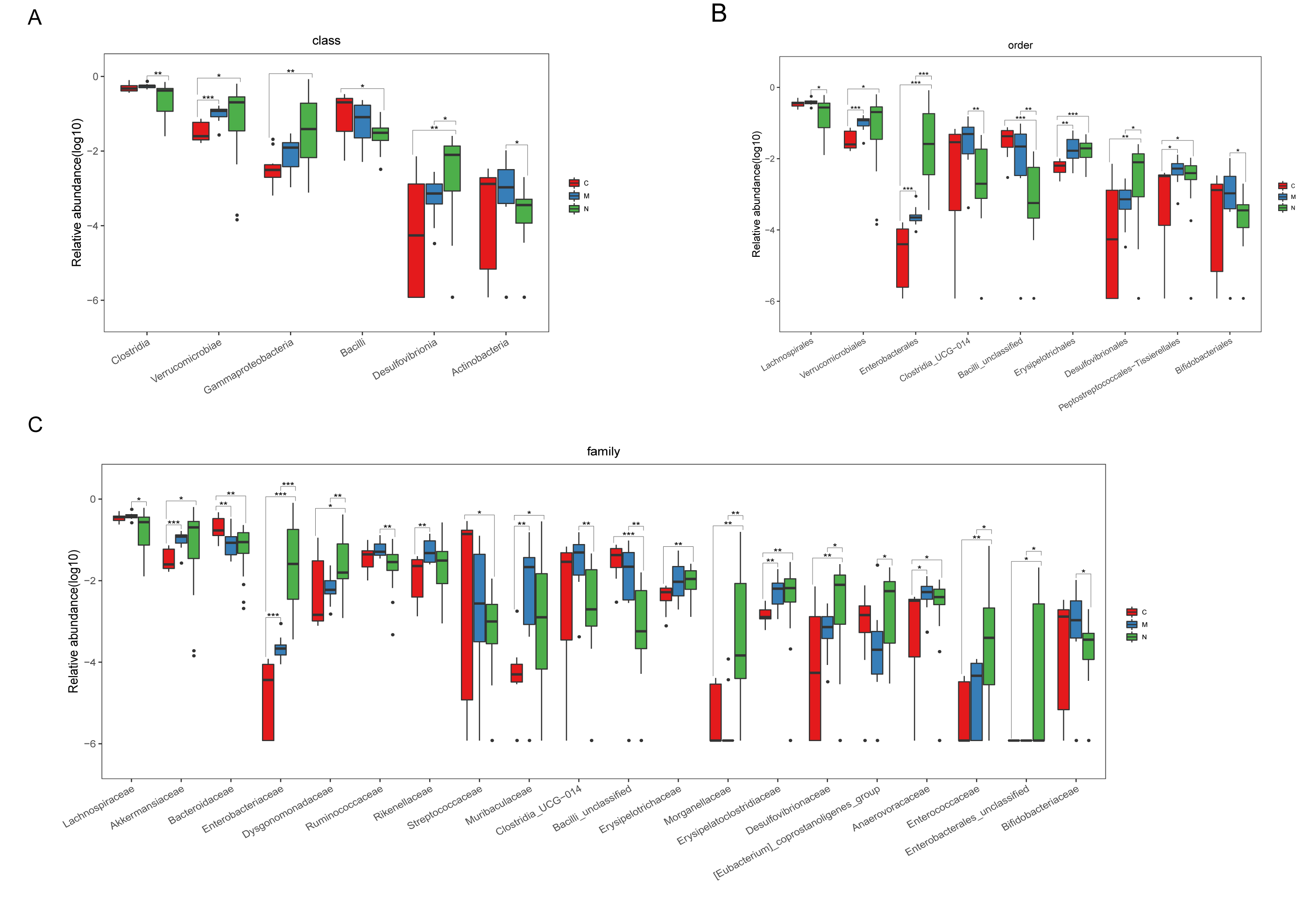

Supplement: Supplementary file 2 [file Image3.TIF]

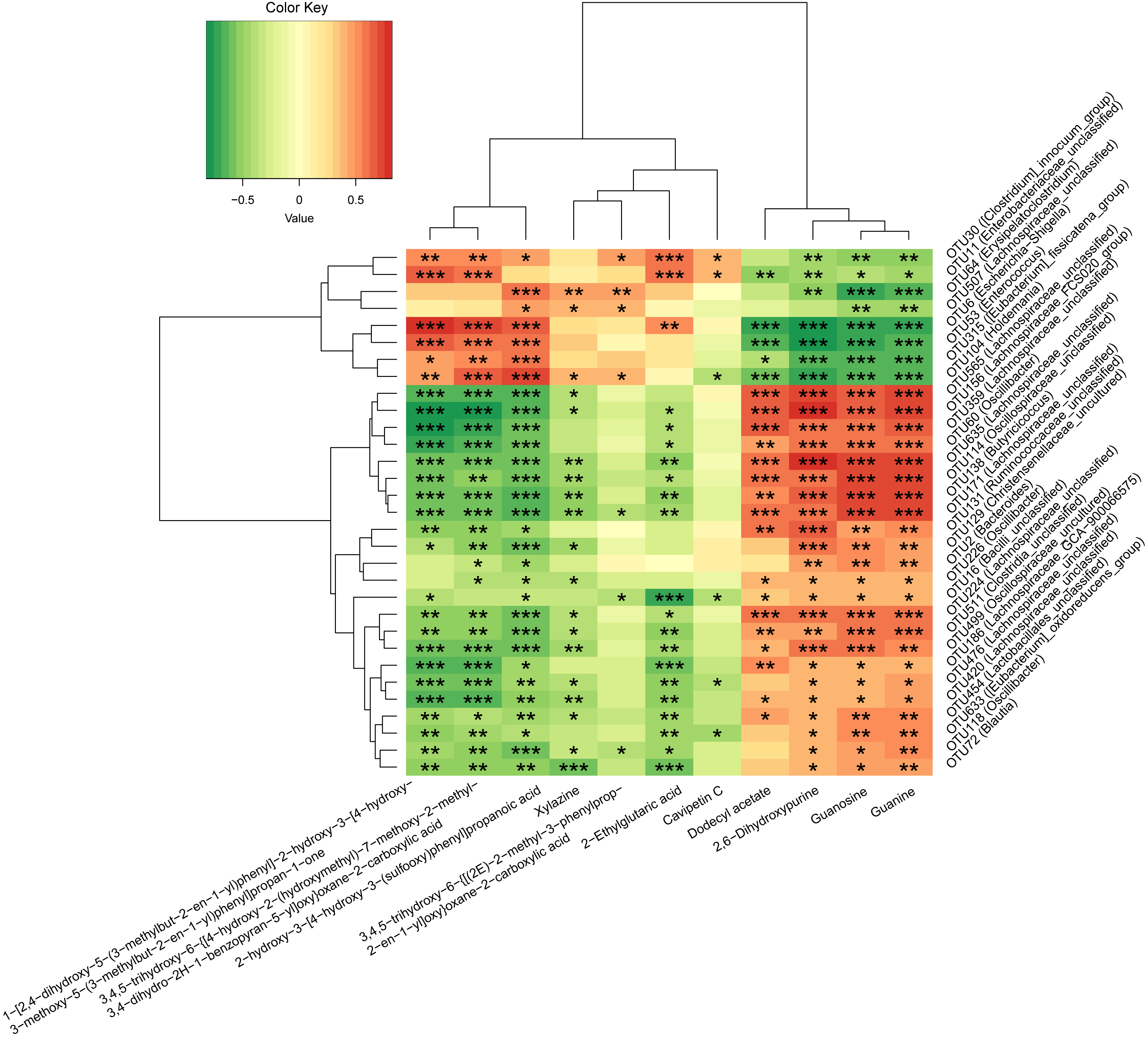

Supplement: Supplementary file 3 [file Image4.TIF]

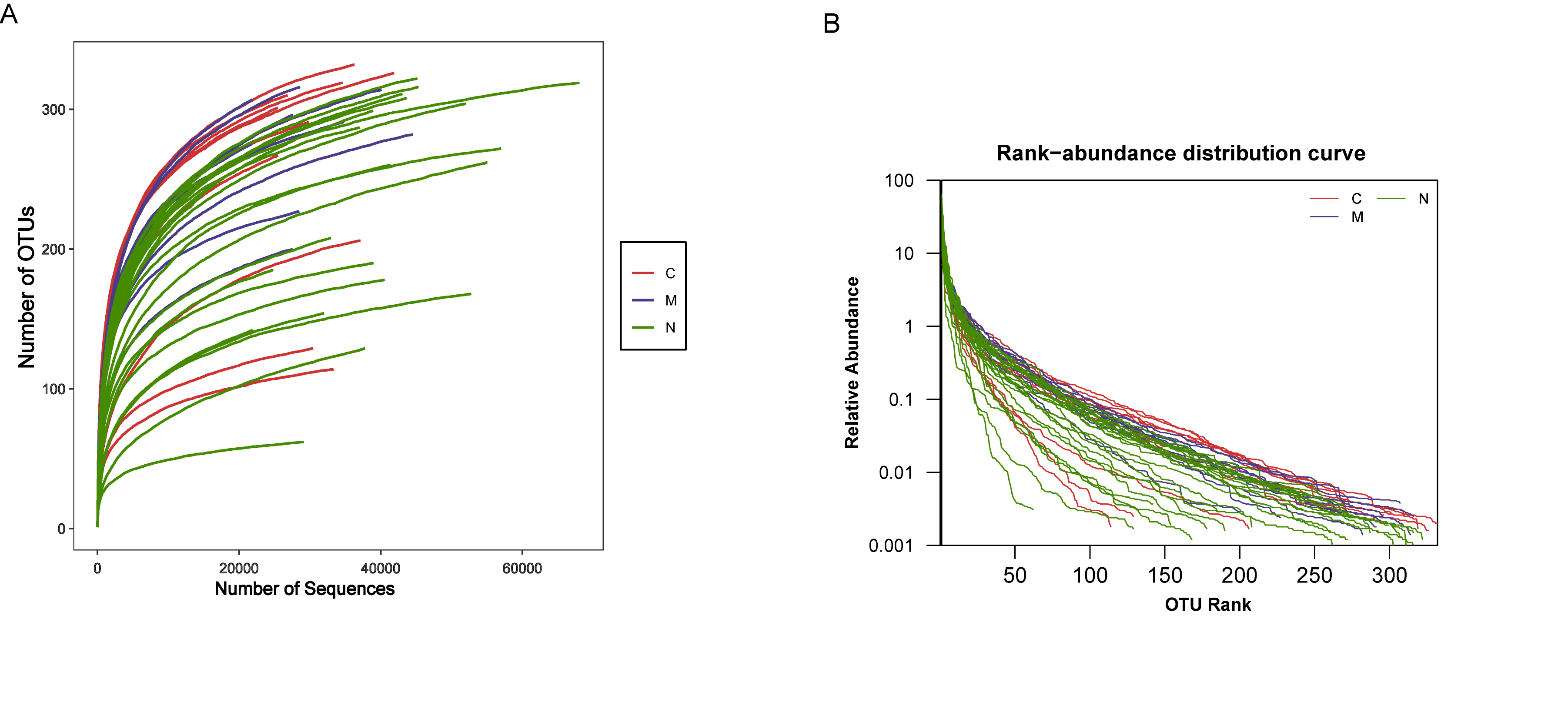

Supplement: Supplementary file 4 [file Image2.TIF]

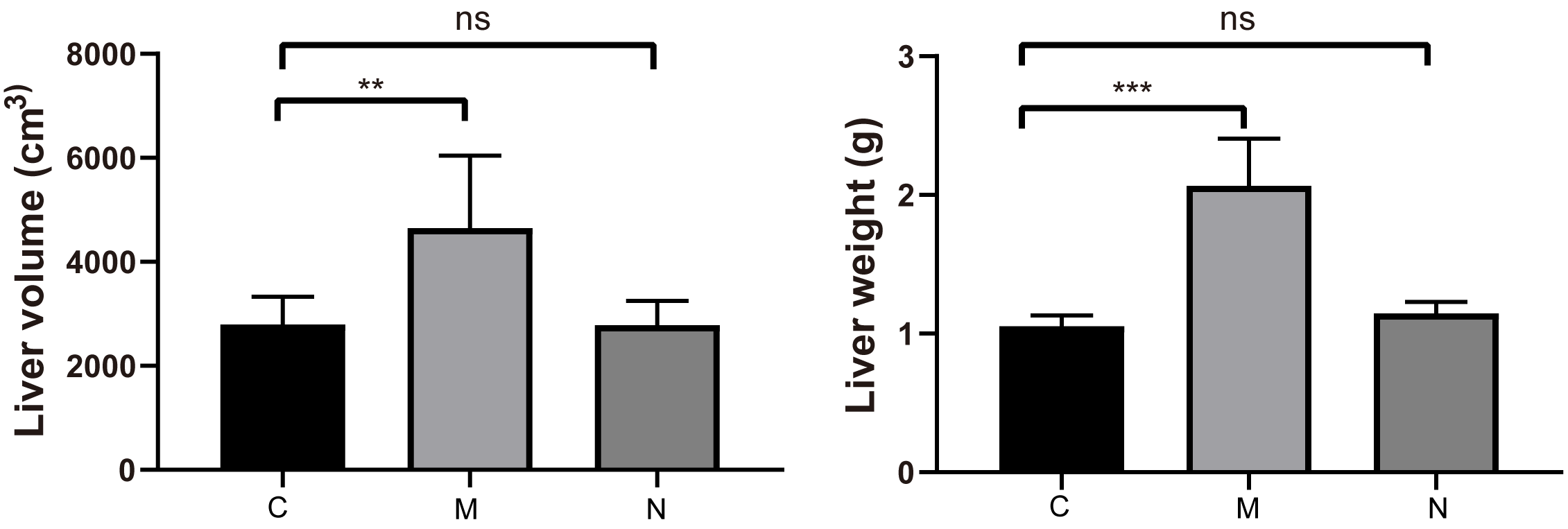

Supplement: Supplementary file 5 [file Image1.TIF]

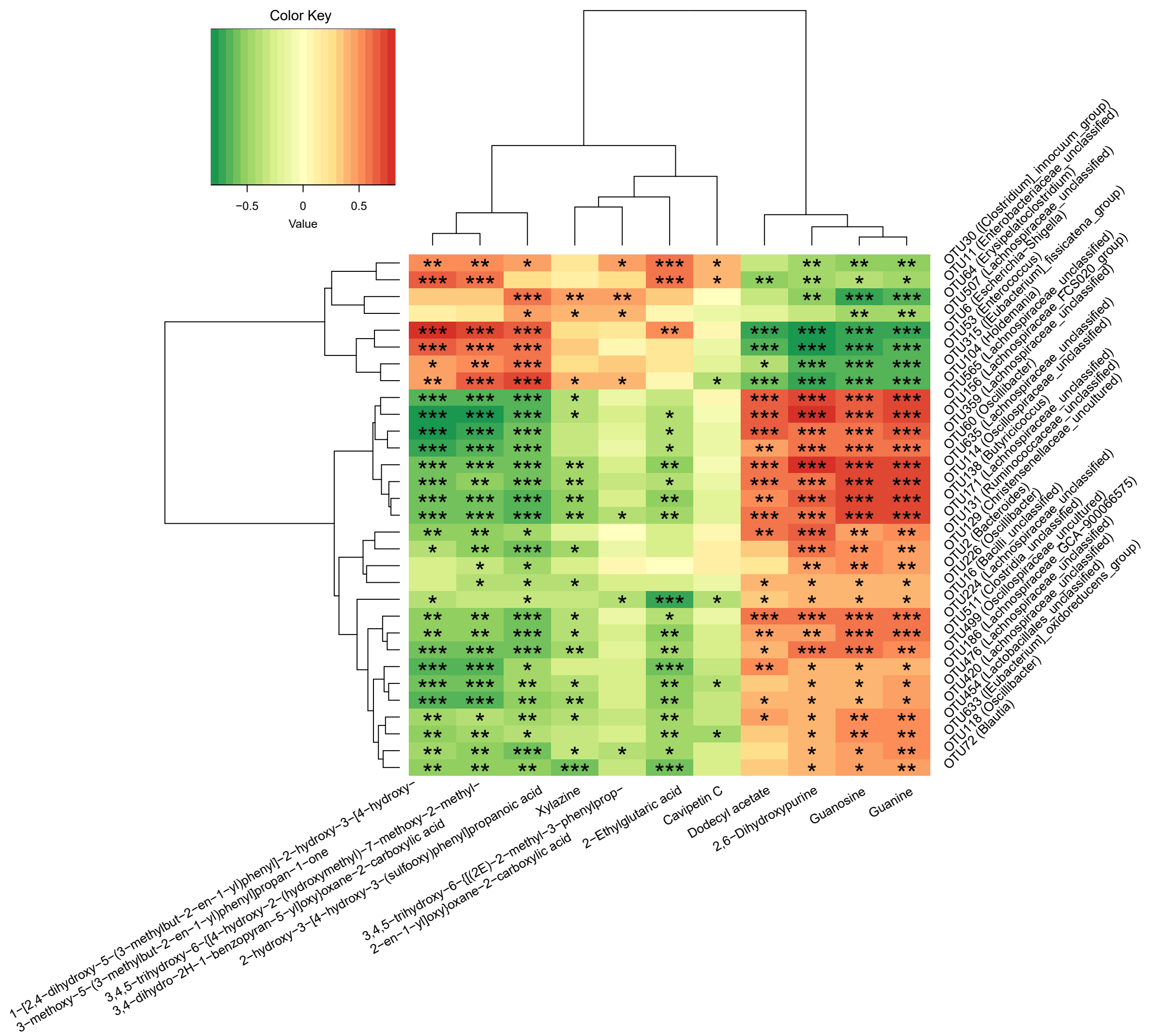

Supplement: Supplementary file 6 [file DataSheet3.PDF]

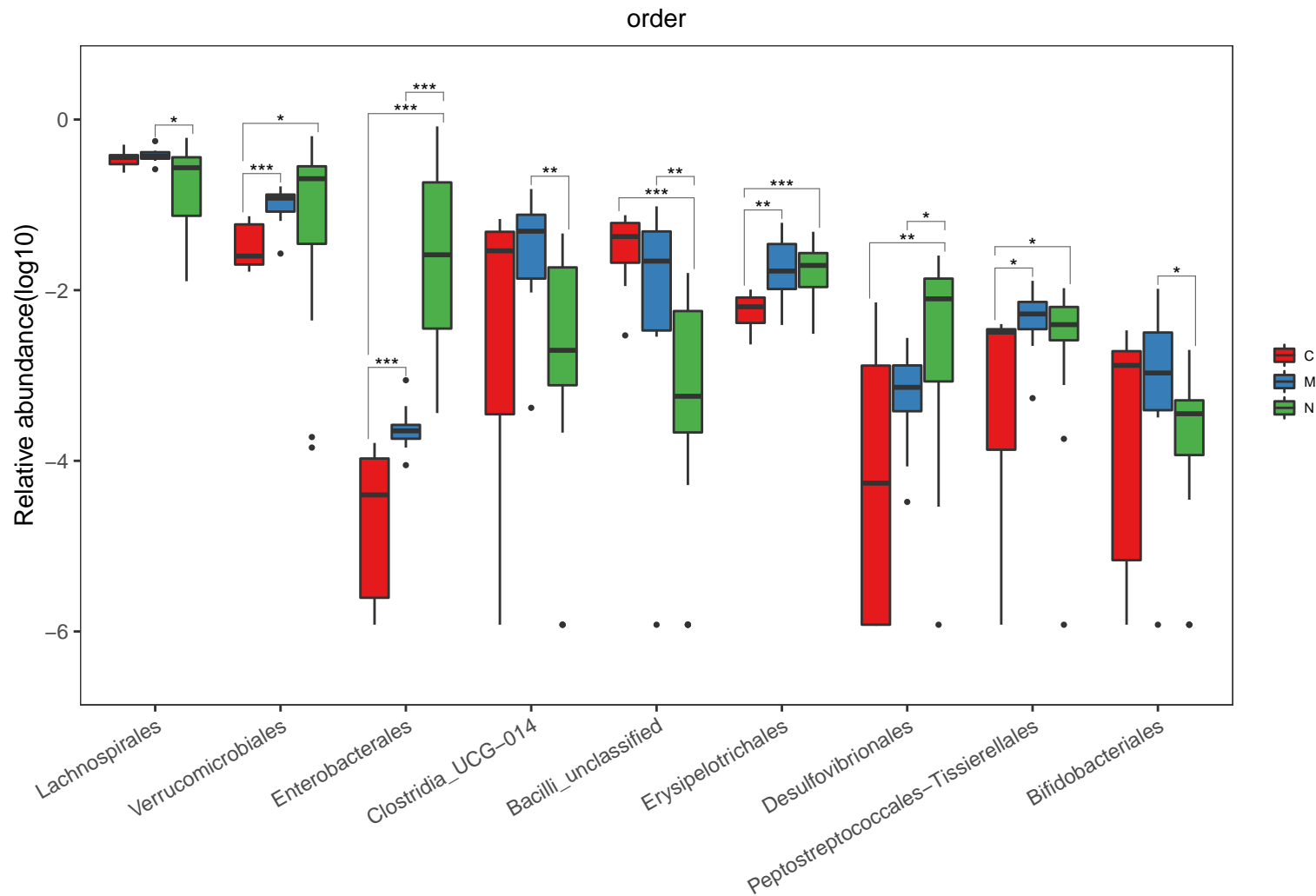

Supplement: Supplementary file 7 [file DataSheet1.PDF]
